# New Neotropical species of *Chimarra* (Trichoptera, Philopotamidae)

**DOI:** 10.3897/zookeys.184.2911

**Published:** 2012-04-21

**Authors:** Roger J. Blahnik, Ralph W. Holzenthal

**Affiliations:** 1 Department of Entomology, University of Minnesota, 1980 Folwell Ave., 219 Hodson Hall, St. Paul, Minnesota, 55108, USA

**Keywords:** Trichoptera, Philopotamidae, *Chimarra*, *Chimarrita*, *Otarrha*, new species, caddisfly, Neotropics

## Abstract

Ten new Neotropical species of *Chimarra* are described in the subgenera *Chimarra*, *Chimarrita*, and *Otarrha*. New species in the subgenus *Chimarra* include, in the *Chimarra ortiziana* group: *Chimarra calori* **sp. n.** (southeastern Brazil) and *Chimarra onchyrhina* **sp. n.** (Venezuela); in the *Chimarra picea* group: *Chimarra inchoata* **sp. n.** (Venezuela), *Chimarra nicehuh* **sp. n.** (Venezuela), and *Chimarra sunima* **sp. n.** (Colombia); and in the *Chimarra poolei* group: *Chimarra cauca* **sp. n.** (Colombia) and *Chimarra desirae* **sp. n.** (Bolivia). New species in the subgenus *Chimarrita* include, in the *Chimarra simpliciforma* group: *Chimarra curvipenis* **sp. n.** (SE Brazil) and *Chimarra latiforceps* **sp. n.** (SE Brazil). A single new species in the subgenus *Otarrha* is also described: *Chimarra soroa* **sp. n.** (Cuba). Males and females for all of the new species are illustrated, except for *Chimarra desirae*, for which female specimens were unavailable. Additionally, the female of *Chimarra (Chimarrita) camella*, which was previously unknown, is illustrated.

## Introduction

The genus *Chimarra* Stephens, 1829, with about 700 species worldwide, is the second largest genus in the order Trichoptera (following *Rhyacophila* Pictet, 1834, with about 750 species), and is nearly cosmopolitan in distribution. Many additional species are either known or likely to be described from material existing in collections. Currently 4 subgenera are recognized, *Chimarra* Stephens, 1829; *Curgia* Walker, 1860; *Chimarrita* Blahnik, 1997; and *Otarrha* Blahnik, 2002. All of these occur in the New World, with the latter 3 subgenera restricted to the New World, primarily in the Neotropical region. Collectively, 240 species of *Chimarra* are recognized for the New World, or somewhat over a third of the world fauna. This entire New World fauna has been relatively recently revised: the subgenus *Chimarra* for Eastern United States by Lago and Harris (1987), Neotropical and Mexican species of the subgenus *Chimarra*, including species from western United States, by Blahnik (1998), the subgenus *Curgia* by Flint (1998), the subgenus *Chimarrita* by Blahnik (1997), and the subgenus *Otarrha* by Blahnik (2002). Two additional species were treated as *incertae sedis* as to subgenus in the paper on *Otarrha* by Blahnik (2002). Only 2 new Neotropical species have been described since these works appeared: *Chimarra chimalapa*, in the subgenus *Chimarra*, described by Bueno-Soria et al. (2001) from Mexico, and *Chimarra paucispina*, in the subgenus *Curgia*, described by Santos and Nessimian (2009) from Brazil. This paper includes new species in the subgenera *Chimarra*, *Chimarrita*, and *Otarrha*, based on material collected or curated subsequent to these revisions. All of these species fit within the general phylogenetic infrastructure established for these subgenera by Blahnik (1997, 1998, 2002), and the sister taxon relationships discussed here are based on those analyses. New species in the subgenus *Chimarra* fall within the *Chimarra ortiziana*, *Chimarra picea*, and *Chimarra poolei* groups, which also represent several of the most species diverse of the 17 New World species groups recognized by Blahnik (1998). A key to species groups and previously described species can be found in the same work (Blahnik 1998: 18). The 2 new species in the subgenus *Chimarrita* fall in the *Chimarra simpliciforma* group, and individually represent closely related sister taxa to 2 of the described species. The new species in the subgenus *Otarrha* from Cuba falls within the Greater Antillean lineage of species in that subgenus and is a probable sister taxon to *Chimarra garciai* Botosaneanu, 1980, also described from Cuba.

## Materials and methods

Methodology follows that used by Blahnik (1998) for *Chimarra* and as discussed by Blahnik and Holzenthal (2004) for caddisflies in general. Additionally (when possible), the lactic acid method (Blahnik et al. 2007) was used to fully inflate the endotheca of individual specimens of most species. Because of the very elongate, narrow endotheca often found in *Chimarra (Chimarra)*, the method was most successful on specimens in which the endotheca was already partially inflated. Illustrations were drawn with use of an optical grid, scanned, and illustrated in Adobe Illustrator CS®. Terminology follows that established by Blahnik (1997, 1998, 2002) for the respective subgenera of *Chimarra*. Descriptions for new species were formulated to closely follow those in the corresponding publications.

Each pinned specimen, or lot of specimens in alcohol, examined during the study, was affixed with a barcode label (4 mil polyester, 8 × 14 mm, code 49) with a unique alphanumeric sequence preceded with the prefix UMSP. The prefix is not meant to imply ownership by the University of Minnesota Collection, but only to indicate that the specimen was databased in that collection. Specimen taxonomic and collection data are stored in Biota® (v. 2.0, Sinauer Associates, Inc.) (Colwell 2003). Specimen barcode label information is included for holotypes in the list of material examined. A detailed list of all material examined, including individual barcode numbers, is maintained at UMSP and can be provided on request.

Holotypes are deposited in the collections of the University of Minnesota, St. Paul, Minnesota, USA (UMSP), the National Museum of Natural History, Smithsonian Institution, Washington DC, USA (NMNH), the Museu de Zoologia, Universidade de São Paulo, São Paulo, Brazil (MZUSP), and the Museo de Historia Natural Noel Kempff Mercado, Santa Cruz de la Sierra, Bolivia (UASC), as designated in the species descriptions. Paratypes are deposited in the same institutions, and also in the Museo del Instituto de Zoología Agrícola, Universidad Central de Venezuela, Maracay, Venezuela (MIZA).

## Species descriptions

### *Chimarra (Chimarra) ortiziana* group

Blahnik (1998) recognized 12 species in this group, distributed from Mexico to Ecuador and Venezuela. The new species in this group from southeastern Brazil represents a significant range extension for the group. The group is recognized primarily on the structures of the male genitalia, and particularly the general shape of the inferior appendages, which is very consistent among the described species, relatively short, with a prominent dorsal thumb-like process, which is typically somewhat mesally curved. The group is closely related to several other species groups, including the *Chimarra amica*, *Chimarra dentosa*, and *Chimarra bidens* groups (and possibly also the *Chimarra cornuta* and *Chimarra virgencita* groups), based especially on the structure of the phallotremal sclerite complex and general similarity in structure of the inferior appendages. Of these, only the *Chimarra bidens* group, with 6 described species, has more than 1 or 2 species. The new species described here represent sister species to 2 of the described species in the *Chimarra ortiziana* group, based both on characters from the original analysis and also overall similarity.

#### Chimarra (Chimarra) calori

Blahnik & Holzenthal sp. n.

urn:lsid:zoobank.org:act:06789F2A-D430-442F-9DEB-9AC4DF5889FB

http://species-id.net/wiki/Chimarra_calori

Figs 1A–F 8

##### Description.

This species is most closely related to *Chimarra gondela* Flint, 1974, based on characters of the male and female genitalia. Character synapomorphies include, especially, the general shape of the lateral lobes tergum X, which are narrow overall and each of which has a raised basodorsal protuberance and basally located sensilla; and also the structure of the phallic apparatus, which includes a short, curved, sclerotized apicoventral endothecal spine in both species, in addition to the usual pair of basal endothecal spines. The lateral lobes in *Chimarra calori* differ in that they are not quite as narrow or strongly sclerotized apically as in *Chimarra gondela*. The most significant difference is in the dorsal thumb-like projections of the inferior appendages, which in *Chimarra calori* are bluntly rounded apically and closely resemble those of many other species in the *Chimarra ortiziana* group, but in *Chimarra gondela* are acute apically and very narrowed and bent.

*Adult*. Forewing length (male) 4.4–4.9 mm, (female) 4.7–5.5 mm. Cuticle of head and thorax very dark, nearly black, setae of anteromesal and frontal setal warts whitish, setae of other setal warts brownish-black, grizzled (a few setae gray or with apices grayish), femora brown, otherwise color nearly uniformly brownish-black (fuscous), including appendages and antennae. Postocular parietal sclerite short (not greatly extended behind eye). Second segment of maxillary palp slightly shorter than segment 3. Male protarsal claws enlarged, asymmetrical in size and shape, outer claw much larger, twisted, nearly linear apically.

*Male genitalia*. Abdominal segment IX, in lateral view, with pronounced sinuous extension of anteroventral margin and with distinct apodemes from the anterodorsal margin; posteroventral process moderately elongate, subtriangular, relatively wide basally, subacute apically. Tergum X with mesal lobe membranous; lateral lobes sclerotized, each bearing short conical lateral projection basally, with 2 sensilla, lobes relatively narrow, as viewed laterally, sclerotized along dorsal margins; apices of paired lobes diverging, as viewed dorsally, forming small, angular projections. Preanal appendage short, knob-like. Inferior appendage, in lateral view, with subquadrate base, dorsally with flattened, thumb-like extension, flexed caudally as viewed laterally, and mesally as viewed caudally, apex of thumb-like projection rounded, mesal surface with sclerotized ridge. Phallotheca with apicoventral extension; endotheca with minute spines and sclerous region formed into short curved spine, endothecal spines 2, subequal in length, relatively short. Phallotremal sclerite complex composed of elongate rod and ring structure, with associated pair of wishbone-like sclerites from anteroventral margin.

*Female genitalia*. Sternum IX with ventral lobes broadly truncate apically, laterally with distinct cupped clasper receptacles. Vaginal apparatus, as viewed ventrally, with transverse apicodorsal sclerite, somewhat narrowed mesally and forming slightly projecting lobes laterally; apicoventrally with paired, subquadrate sclerites, each with narrow, sclerotized anterior extension, projecting onto lateral margin of vaginal apparatus; lateral margins rounded and membranous; vaginal apparatus anteriorly with deflexed, cup-like sclerite and paired preapical dorsal and ventral sclerites, dorsal pair elongate, narrow, ventral pair shorter, subtriangular.

##### Holotype

**, male** (pinned) (UMSP000120377)**: BRAZIL: São Paulo:** Altinópolis, Cachoeira Dos Macacos, 20°55.380'S, 47°22.758'W, 759 m, 18.xi.2003, Holzenthal, Paprocki, Calor (MZUSP).

**Figure 1.**
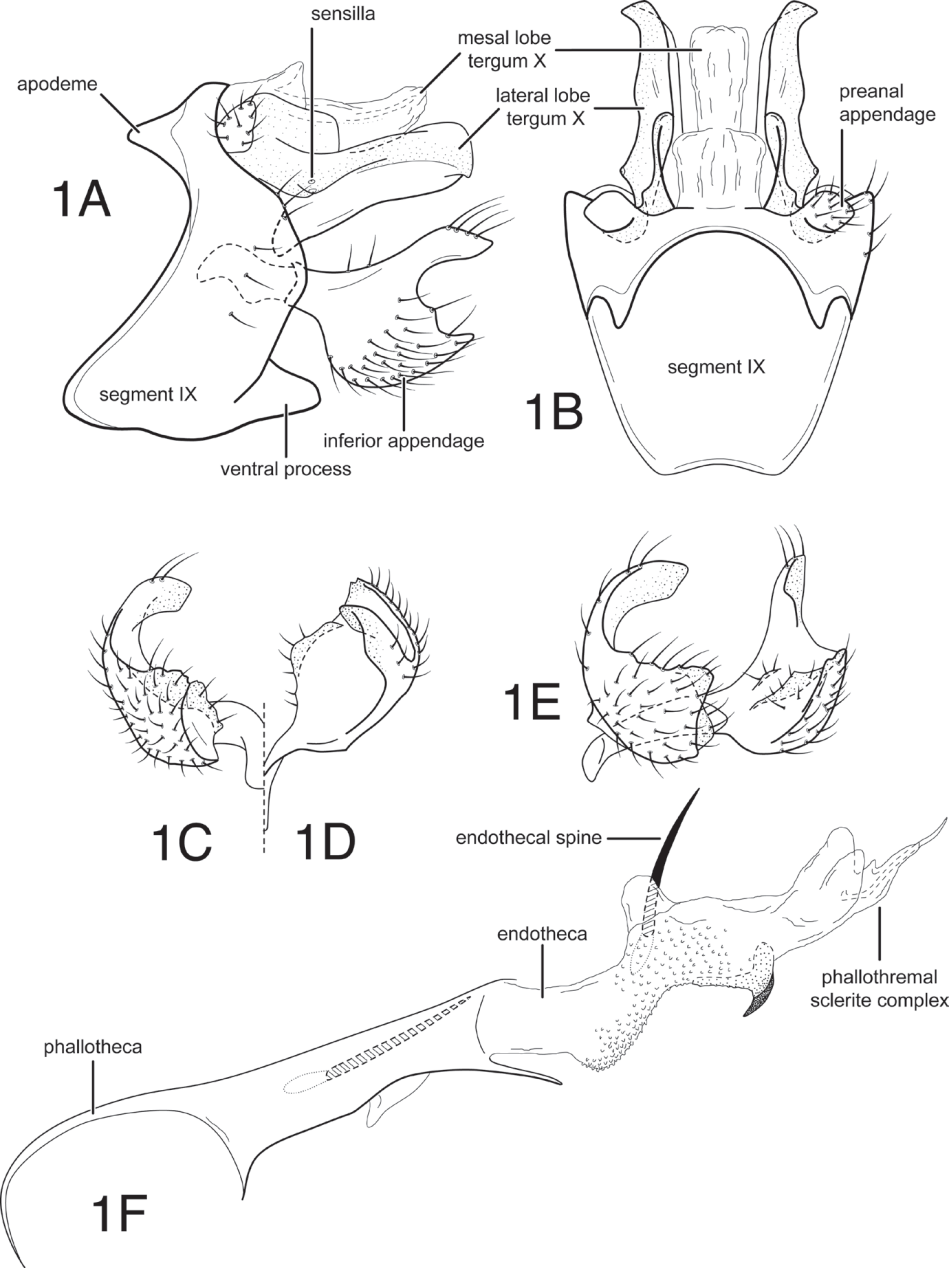
*Chimarra (Chimarra) calori* sp. n. Male genitalia: **A** lateral **B** segment IX and tergum X, dorsal **C** inferior appendage, caudal **D** inferior appendage, dorsal **E** inferior appendages, oblique lateral **F** phallic apparatus, lateral.

##### Paratypes.

**BRAZIL:** **Minas Gerais:** spring trib. to Rio Macauba, near Pandeiros, 15°28.637'S, 44°44.627'W, 525 m, 17.xi.2001, Paprocki & Blahnik, 3 males, 23 females (pinned) (UMSP); Parque Nacional Peruaçu, Rio Peruaçu, 15°06.674'S, 44°14.487'W, 590 m, 16.xi.2001, Holzenthal, Paprocki, Blahnik, Amarante, 13 males, 11 females (pinned) (UMSP) (MZUSP) (NMNH), 1 male (alcohol) (UMSP); Rio Guanhães, downstream from Salto Grande dam, 19°06.289'S, 42°42.635'W, 20.x.1998, Paprocki, 1 male, 1 female (pinned) (UMSP**); São Paulo:** same data as holotype, 3 males, 2 females (pinned) (UMSP).

##### Etymology.

This species is named *Chimarra calori*, for Dr. Adolfo Calor, Universidade Federal da Bahia, Brazil, who helped collect the type specimen, in recognition of his contributions to the study of the Neotropical caddisflies.

#### Chimarra (Chimarra) onchyrhina

Blahnik & Holzenthal sp. n.

urn:lsid:zoobank.org:act:E01AAEF0-475A-4AF7-9413-47DB0B33AA1F

http://species-id.net/wiki/Chimarra_onchyrhina

Figs 2A–F 9

##### Description.

This species is very similar to *Chimarra platyrhina* Flint, 1981, especially in the general shape of the lateral lobes of tergum X, as viewed dorsally. In both species, the lateral lobes of tergum X each have a broadly rounded apicolateral projection. The difference in shape of this projection, however, is distinctive and diagnostic, that of *Chimarra platyrhina* being broad and uniformly rounded, and that of *Chimarra onchyrhina* being somewhat angular laterally. Both species also have a linear cluster of small spines located near the ventral apex of the endotheca, also present in some other species of the *Chimarra ortiziana* group. The latter character is, however, absent in *Chimarra ortiziana*, which otherwise resembles *Chimarra onchyrhina* in the general shape of the apex of the lateral lobes of tergum X. An additional difference separating them is that *Chimarra ortiziana* has the lateral sensilla of tergum X placed on a concavely developed lateral projection. The lateral projections of *Chimarra onchyrhina* and *Chimarra platyrhina* are simple in structure. The female genitalia of *Chimarra onchyrhina* also generally resemble *Chimarra platyrhina*, especially in the shape of apical sclerites of the vaginal apparatus. However the vaginal apparatus has distinctive lateral projections, not present in *Chimarra platyrhina*. The overall similarities of these species, both in male and female genitalia, are indicative of their sister relationship.

*Adult*. Forewing length (male) 3.9–5.0 mm, (female) 4.8–5.5 mm. Cuticle of head and thorax dark brown, setae of anteromesal and frontal setal warts whitish, setae of other setal warts dark brown, grizzled (grayish in part or intermixed), femora brown, otherwise color nearly uniformly brownish-black (fuscous), including appendages and antennae. Postocular parietal sclerite very short (not extended behind eye). Second segment of maxillary palp much shorter than segment 3 (about 2/3 length). Male protarsal claws enlarged, asymmetrical in size and shape, outer claw much larger, twisted, nearly linear apically.

*Male genitalia*. Abdominal segment IX, in lateral view, with pronounced sinuous extension of anteroventral margin and with distinct apodemes from anterodorsal margin; posteroventral process moderately elongate, subtriangular, relatively broad basally, subacute apically. Tergum X membranous mesally, with 2 sclerotized lateral lobes, each bearing short rounded projection laterally in basal portion, with 2 sensilla on tiny projections; terminus of lateral lobe inflated, broadly rounded, but with distinct, obtuse angle on lateral margin. Preanal appendage short, knob-like. Inferior appendage, in lateral view, with rounded to subquadrate base, subacute apically; dorsally with flattened, thumb-like extension, flexed caudally as viewed laterally, and mesally as viewed caudally; apex of thumb-like projection rounded. Phallotheca with apicoventral margin projecting; endotheca forming collar of minute spines in apical 1/2, slightly ballooned laterally, apicoventrally with sclerotized region possessing array of short spines, endothecal spines 2, very short, subequal in length. Phallotremal sclerite complex composed of elongate rod and ring structure with associated pair of wishbone-like sclerites from anteroventral margin.

*Female genitalia*. Sternum IX with ventral lobes broadly truncate apically. Vaginal apparatus, as viewed ventrally, with paired, subtruncate, lightly sclerotized apicoventral sclerites; laterally with short projecting sclerotized lobes on either side, each with short, digitate apical projection; vaginal apparatus anteriorly with deflexed, cup-like sclerite and elongate, paired, dorsolateral and ventrolateral sclerites, these extending more than ½ length of vaginal apparatus.

**Figure 2:**
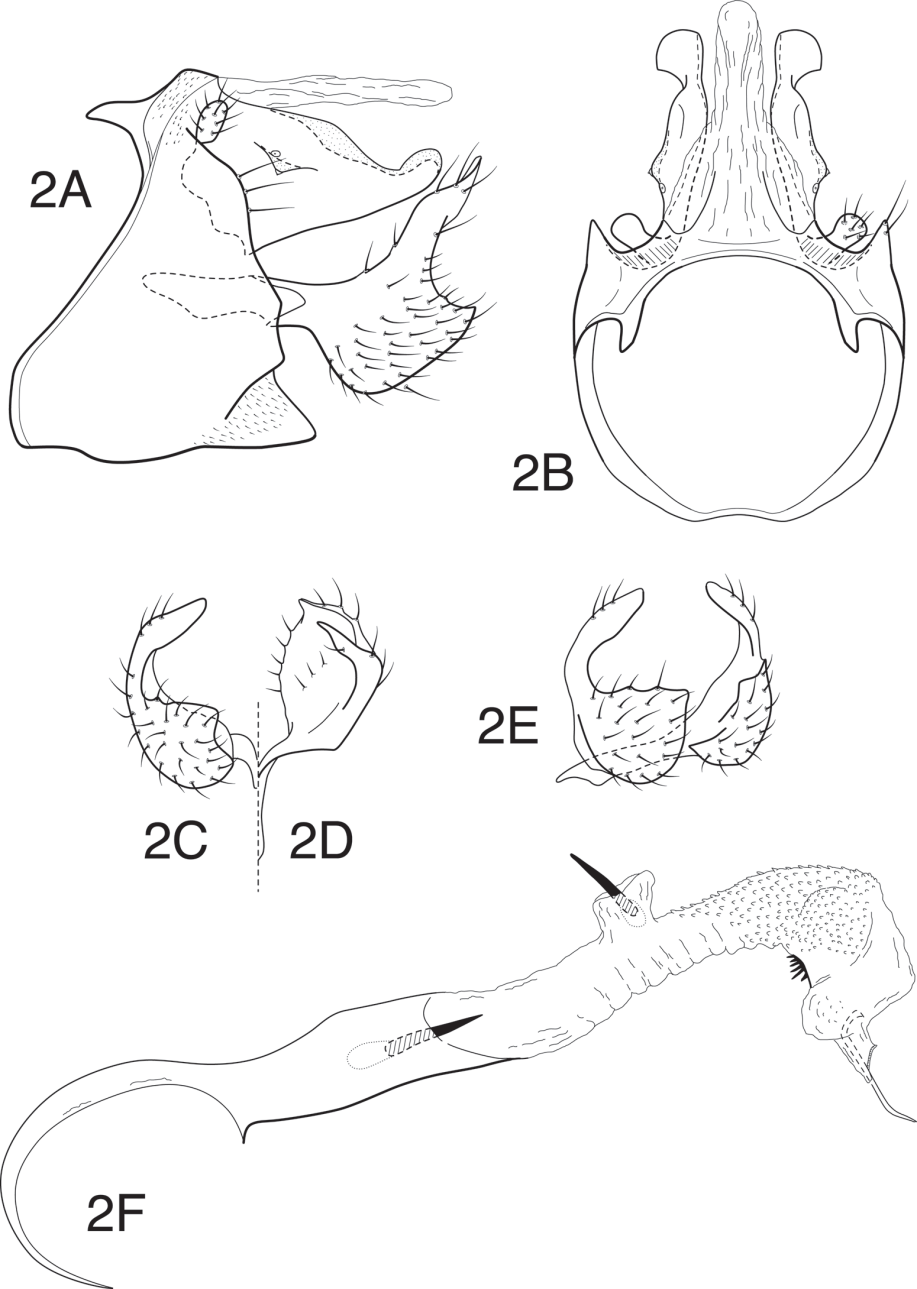
**.** *Chimarra (Chimarra) onchyrhina* sp. n. Male genitalia: **A** lateral **B** segment IX and tergum X, dorsal **C** inferior appendage, caudal **D** inferior appendage, dorsal **E** inferior appendages, oblique lateral **F** phallic apparatus, lateral.

##### Holotype

**, male** (pinned) (UMSP000026958)**:** **VENEZUELA:** **Sucre:** Península de Paria, Puerto Viejo, “Río el Pozo”, 10°43.073'N, 62°28.569'W, 20 m, 3.iv.1995, Holzenthal, Flint, Cressa (UMSP).

##### Paratypes.

**VENEZUELA: Falcón:** Quebrada El Charo at cataratas, 10°46.771'N, 69°12.174'W, 425 m, Holzenthal, Blahnik, Paprocki, Cressa, 3 males (alcohol) (UMSP); **Monagas:** Guachero Cave N.P., 10°10.322'N, 63°33.315'W, 1110 m, 20–21.vii.2010, Holzenthal, Thomson, Cressa, 4 males, 6 females (pinned) (UMSP); **Sucre:** same data as holotype, 12 males (pinned), 33 males, 20 females (alcohol) (UMSP); Península de Paria, Santa Isabel, Río Santa Isabel, 10°44.294'N, 62°38.954'W, 20 m, 4.iv.1995, Holzenthal, Flint, Cressa, 9 males, 1 female (pinned), 28 males, 45 females (alcohol) (UMSP); Península de Paria, Puerto Viejo, Río Puerto Viejo, 10°43.137'N, 62°28.743'W, 15 m, 2.iv.1995, Holzenthal, Flint, Cressa, 3 males (pinned), 20 males, 60 females (alcohol) (UMSP); Parque Nacional Península de Paria, Río San Francisco, 10°42.713'N, 62°00.066'W, 10 m, 1.iv.1995, Holzenthal, Flint, Cressa, 2 males (pinned) (UMSP); Parque Nacional Península de Paria, Uquire, Río La Viuda, 10°42.830'N, 61°57.661'W, 15 m, 30.iii–1.iv.1995, Holzenthal, Flint, Cressa, 15 males (pinned), 18 males, 43 females (alcohol) (UMSP) (NMNH) (MIZA); Quebrada Zapateral, 1.5 km SE Las Piedras de Cocollar, 10°09.753'N, 63°47.587'W, 810 m, 9.iv.1995, Holzenthal & Flint, 2 males, 1 female (alcohol) (NMNH); Río Cocollar, 1.5 km SE Las Piedras de Cocollar, 10°09.617'N, 63°47.605'W, 810 m, 7–8.iv.1995, Holzenthal & Flint, 12 males, 10 females (alcohol) (MIZA).

##### Etymology.

This species is named *Chimarra onchyrhina* from the Greek words *onkos*, meaning a hook or angle, and *rhinos*, a nose, and referring to the slightly angled lateral process on tergum X of this species, which distinguishes it from the more uniformly rounded process in *Chimarra platyrhina*.

### *Chimarra (Chimarra) picea* group

Blahnik (1998) recognized 11 species in this group, distributed from lower Central America to Ecuador and Venezuela. The species all have very similar, though somewhat differently developed, inferior appendages (very short with elongate lateral setae and small, sclerotized, strongly mesally curved dorsomesal processes, not generally evident in lateral view), and are especially diagnosed by differences in the shape of the lateral lobes of tergum X and their sensilla-bearing protuberances. The 3 new species recognized here are similar in general characters to other described species and bring the total number of species in the group to 14.

#### Chimarra (Chimarra) inchoata

Blahnik & Holzenthal sp. n.

urn:lsid:zoobank.org:act:E6B24613-4FFD-4066-9311-0E8B066AD613

http://species-id.net/wiki/Chimarra_inchoata

Figs 3A–G 10

##### Description.

This species is most closely related to *Chimarra creagra* Flint, 1981 and *Chimarra paracreagra* Blahnik, 1998, based on the similarly developed, sclerotized, dorsolateral margins of the mesal lobe of tergum X. In *Chimarra inchoata*, however, this structure is not as strongly sclerotized, and while somewhat curved apically, does not form the strongly recurved and acutely hooked projections found in the two described species. Additionally, the sclerotized basodorsal, mesally curved, thumb-like processes of the inferior appendage are distinctly evident even in lateral view, and the lateral lobes of tergum X are shorter and have much more broadly rounded lateral sensilla-bearing processes.

*Adult*. Forewing length (male) 3.7–5.1 mm, (female) 4.4–5.8 mm. Cuticle of head and thorax dark brown, setae of anteromesal and frontal setal warts light brown or brownish-white, setae of other setal warts dark brown, grizzled (grayish at apices or intermixed with grayish setae), femora brown, otherwise color nearly uniformly dark brown or brownish-black (fuscous), including appendages and antennae. Postocular parietal sclerite elongate (extended behind eye). Second segment of maxillary palp much shorter than segment 3 (less than 2/3 length). Male protarsal claws enlarged, asymmetrical in size and shape, outer claw much larger, twisted, nearly linear apically.

*Male genitalia*. Abdominal segment IX, in lateral view, with pronounced linear extension of anteroventral margin and with distinct, enlarged apodemes from anterodorsal margin; posteroventral process prominent, subtriangular, broad basally, subacute apically. Tergum X with mesal lobe membranous and incised mesally, lightly sclerotized laterally, forming bluntly rounded, dorsally recurved projections; lateral lobes sclerotized, relatively short, tapering apically, each bearing broadly rounded projection laterally with 2 sensilla. Preanal appendage short, knob-like. Inferior appendage short, subtruncate, with elongate marginal setae, dorsally with short, bluntly rounded, mesally curved process, distinctly visible in both lateral and dorsal views. Phallotheca with acute apicoventral projection; endotheca membranous with scattered, minute spines, these slightly longer preapically on ventral margin, endotheca dorsally with slightly ballooned lateral projections, endothecal spines 2, basal one moderately elongate and curved, apical one very elongate and nearly straight. Phallotremal sclerite complex composed of rod and ring structure, rod relatively elongate, ring reclinate.

*Female genitalia*. Sternum IX with ventral lobes truncate apically, laterally with small rounded clasper receptacles. Vaginal apparatus relatively membranous apically, with narrow, transverse preapical sclerite dorsally; ventrally with elongate, narrow, membranous lobe extending from apex to about ½ length, lobe with small sclerite at apex; vaginal apparatus laterally with pair of small sclerites at about midlength; anteriorly with deflexed, cup-like sclerite.

**Figure 3.**
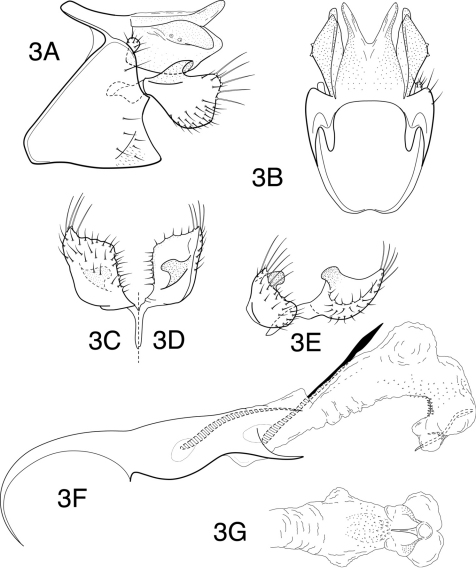
*Chimarra (Chimarra) inchoata* sp. n. Male genitalia: **A** lateral **B** segment IX and tergum X, dorsal **C** inferior appendage, ventral **D** inferior appendage, dorsal **E** inferior appendages, oblique lateral **F** phallic apparatus, lateral **G** apex of endotheca, ventral.

##### Holotype

**, male** (alcohol) (UMSP000026863)**:** **VENEZUELA:** **Sucre:** Península de Paria, Puerto Viejo, Río Puerto Viejo, 10°43.137'N, 62°28.743'W, 15 m, 2.iv.1995, Holzenthal, Flint, Cressa (UMSP).

##### Paratypes.

**VENEZUELA:** **Monagas:** Guachero Cave N.P., 10°10.322'N, 63°33.315'W, 1110 m, 20–21.vii.2010, Holzenthal, Thomson, Cressa, 32 males, 10 females (pinned) (UMSP); **Sucre:** Río Cocollar, 1.5 km SE Las Piedras de Cocollar, 10°09.617'N, 63°47.605'W, 810 m, 7–8.iv.1995, Holzenthal & Flint, 26 males (pinned) 201 males, 101 females (alcohol) (UMSP) (MIZA); Parque Nacional Península de Paria, Río San Francisco, 10°42.713'N, 62°00.066'W, 10 m, 1.iv.1995, Holzenthal, Flint, Cressa, 1 male (pinned) (UMSP); Parque Nacional Península de Paria, Uquire, Río La Viuda, 10°42.830'N, 61°57.661'W, 15 m, 30.iii-1.iv.1995, Holzenthal, Flint, Cressa, 25 males, 1 female (pinned), 81 males, 43 females (alcohol) (UMSP) (MIZA); Península de Paria, Puerto Viejo, “Río el Pozo”, 10°43.073'N, 62°28.569'W, 20 m, 3.iv.1995, Holzenthal, Flint, Cressa, 16 males (pinned), 94 males, 18 females (alcohol) (UMSP); Península de Paria, Puerto Viejo, Río Puerto Viejo, 10°43.137'N, 62°28.743'W, 15 m, 2.iv.1995, Holzenthal, Flint, Cressa, 5 males, 2 females (pinned), 86 males, 7 females (alcohol) (UMSP) (NMNH); Península de Paria, Santa Isabel, Río Santa Isabel, 10°44.294'N, 62°38.954'W, 20 m, 4.iv.1995, Holzenthal, Flint, Cressa, 17 males (pinned), 55 males, 36 females (alcohol) (UMSP); Quebrada Zapateral, 1.5 km SE Las Piedras de Cocollar, 10°09.753'N, 63°47.587'W, 810 m, 9.iv.1995, Holzenthal & Flint, 7 males (pinned), 70 males, 24 females (alcohol) (UMSP).

##### Etymology.

This species is named *Chimarra inchoata* from the Latin word *incohatus* (or *inchoatus*), meaning only begun, incipient, or incomplete, and referring to the development of the lateral lobes of tergum X in this species, which are only suggestively sclerotized as compared to the strongly sclerotized, hooked processes in *Chimarra creagra* and *Chimarra paracreagra*.

#### Chimarra (Chimarra) nicehuh

Blahnik & Holzenthal sp. n.

urn:lsid:zoobank.org:act:5BE7A6B5-3B82-49E6-AE84-812DAA43C25D

http://species-id.net/wiki/Chimarra_nicehuh

Figs 4A–F 11

##### Description. 

This is perhaps one of the most distinctive of the species in the *Chimarra picea* group described to date and unlikely to be confused with any other described species. Especially distinctive is the shape of the lateral lobes of tergum X, which are short, but broad apically, as viewed laterally, each with a small, obliquely oriented, lateral sensilla-bearing processes. Like the previous species, it has the lateral margins of the mesal lobe of tergum X somewhat sclerotized, though not projecting apically. The thumb-like dorsolateral projections of the inferior appendages are especially blunt and strongly curled, not clearly evident in either lateral or dorsal views. Additionally the apicoventral margin of the appendage is recurved, and thus evident as a ridge on the mesal surface, as viewed caudally.

*Adult*. Forewing length (male) 5.1–5.3 mm, (female) 5.5–5.9 mm. Cuticle of head and thorax very dark, nearly black, setae of anteromesal and frontal setal warts brown or brownish-white, setae of other setal warts and tegulae black, grizzled (grayish at apices or intermixed with grayish setae), otherwise color nearly uniformly dark brownish-black (fuscous), including appendages and antennae (femora not or hardly paler). Postocular parietal sclerite elongate (extended behind eye). Second segment of maxillary palp much shorter than segment 3 (less than 2/3 length). Male protarsal claws enlarged, asymmetrical in size and shape, outer claw much larger, twisted, nearly linear apically.

*Male genitalia*. Abdominal segment IX, in lateral view, with pronounced linear extension of anteroventral margin and with distinct, enlarged apodemes from anterodorsal margin; posteroventral process short, broad basally, rounded apically. Tergum X with mesal lobe membranous and weakly incised mesally, lightly sclerotized laterally; lateral lobes sclerotized, relatively short, very broadly truncate apically as viewed laterally, each bearing short, lateral, obliquely flattened projection with 2 sensilla. Preanal appendage short, knob-like. Inferior appendage very short, apicoventral margin weakly projecting and slightly recurved, apically with elongate marginal setae, dorsally with very short, bluntly rounded, mesally curved process, visible in caudal view, but not fully evident in either lateral or dorsal views. Phallotheca with acute apicoventral projection; endotheca membranous, elongate tubular, with numerous minute spines in apical ½, preapically on ventral margin with very short sclerotized spines, endothecal spines 2, basal one elongate, apical one moderately elongate. Phallotremal sclerite complex composed of rod and ring structure, rod relatively elongate, ring reclinate, complex laterally with pair of short sclerites.

*Female genitalia*. Sternum IX rounded apically, laterally with small, broadly rounded clasper receptacles. Vaginal apparatus relatively membranous apically; laterally with conspicuous, projecting preapical sclerites on either side; anteriorly with deflexed, cup-like sclerite.

**Figure 4.**
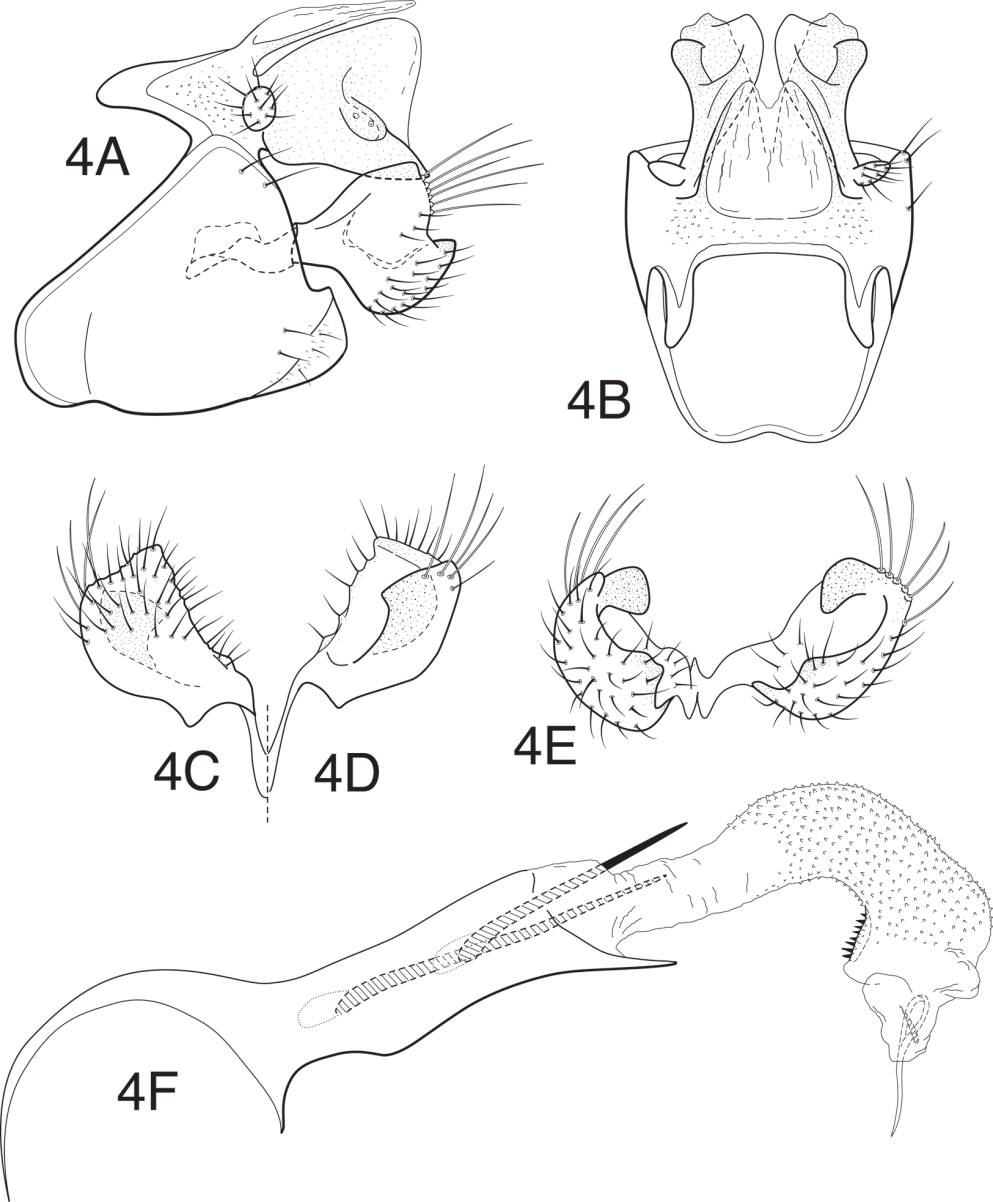
*Chimarra (Chimarra) nicehuh* sp. n. Male genitalia: **A** lateral **B** segment IX and tergum X, dorsal **C** inferior appendage, ventral **D** inferior appendage, dorsal **E** inferior appendages, oblique lateral **F** phallic apparatus, lateral.

##### Holotype

**, male** (pinned) (UMSP000026909)**:** **VENEZUELA:** **Trujillo:** Quebrada Potrerito, 7.5 km NE Boconó, 9°16.435'N, 70°13.102'W, 1530 m, 29-30.iv.1995, Holzenthal, Cressa, Gutic (UMSP).

##### Paratypes.

**VENEZUELA: Lara:** Parque Nacional Dinira, Quebrada Buenos Aires, 9°36.407'N, 70°04.178'W, 1850 m, 18–19.vi.2001, Holzenthal, Blahnik, Paprocki, Cressa, 3 males (pinned) (UMSP); **Trujillo:** same data as holotype, 5 males, 5 females (pinned) (UMSP) (MIZA).

##### Etymology.

The name of this species should be considered an arbitrary combination of letters. It resulted from an observation made by the first author when seeing the genitalia of this distinctive species for the first time, “Nice, huh?”

#### Chimarra (Chimarra) sunima

Blahnik & Holzenthal sp. n.

urn:lsid:zoobank.org:act:C41FE4CB-F191-4B84-8C4A-6F83A03894A2

http://species-id.net/wiki/Chimarra_sunima

Figs 5A–G 12

##### Description. 

This species most closely resembles *Chimarra onima* Flint, 1991, especially in the structure of the lateral sensilla bearing processes of tergum X. In both species the processes have their posterior margins detached and project “ear-like”, as viewed dorsally, although those of *Chimarra sunima* are perhaps not quite so elongate. Differences are found in the basal part of the lateral lobes of tergum X, which in *Chimarra sunima* are more dorsally projecting in lateral view, and in the structure of the inferior appendages. The latter are very distinctive in *Chimarra onima*, with the dorsal margin projecting, so that the dorsomesal processes are clearly apical, as viewed laterally, and the posterior margin concave, whereas in *Chimarra sunima*, the inferior appendages are more or less typical of a number of other species in the group (i.e., *Chimarra tapanti* Blahnik, 1998 or *Chimarra limon* Blahnik, 1998) in that the dorsomesal processes are slightly recessed compared to the elongate, fringing posterolateral setae of the appendage.

*Adult*. Forewing length (male) 3.8–4.5 mm, (female) 4.3–5.2 mm. Cuticle of head and thorax dark brown, setae of anteromesal and frontal setal warts light brown or whitish, setae of other setal warts dark brown, grizzled (grayish in part or intermixed), otherwise color nearly uniformly brownish-black (fuscous), including appendages and antennae. Postocular parietal sclerite elongate (extended behind eye). Second segment of maxillary palp much shorter than segment 3 (less than 2/3 length). Male protarsal claws enlarged, asymmetrical in size and shape, outer claw larger, twisted, nearly linear apically.

*Male genitalia*. Abdominal segment IX, in lateral view, with pronounced linear extension of anteroventral margin and with distinct, enlarged apodemes from anterodorsal margin; posteroventral process prominent, subtriangular, broad basally, subacute apically. Tergum X with mesal lobe membranous; lateral lobes sclerotized, moderately elongate, ventral margin slightly projecting, each lobe bearing broadly truncate, basodorsal projection and dorsoventrally flattened, ear-like lateral projections with 2 sensilla. Preanal appendage short, knob-like. Inferior appendage short, apicoventral margin acutely projecting, apically with elongate marginal setae, dorsally with short, bluntly rounded, mesally curved process, distinctly evident in caudal view, partially evident in lateral and dorsal views. Phallotheca with acute apicoventral projection; endotheca membranous, elongate tubular, with numerous minute spines in apical ½, endothecal spines 2, basal one elongate, apical one short and curved. Phallotremal sclerite complex composed of rod and ring structure, rod relatively elongate, ring reclinate, laterally with short sclerites attached to membranous apical wing-like projections.

*Female genitalia*. Sternum IX with ventral lobes tapering, subacute, extreme apex nearly membranous, laterally with large rounded clasper receptacles. Vaginal apparatus relatively membranous apically, with very narrow, elongate mesal lobe extending from apex to more than ½ length, lobe very lightly sclerotized; laterally with conspicuous, projecting preapical lobes on either side, these largely membranous, but with conspicuous, rounded, premarginal ventral sclerites; anterolateral margins of vaginal apparatus lightly sclerotized; anteriorly with deflexed, cup-like sclerite.

##### Holotype

**, male** (pinned) (UMSP000209321)**: COLOMBIA:** **Valle:** Municipio de Buenaventura, Río Escalerete, frente a casa de “Acua Valle", ca. 15 km SE Cordoba, 3°49'38"N, 76°52'15"W, 200 m, 1.xii.1997, F. Muñoz-Q. et al. (UMSP)

**Figure 5.**
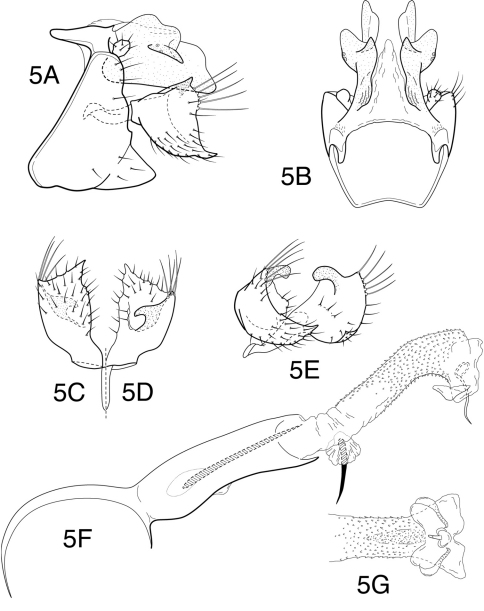
*Chimarra (Chimarra) sunima* sp. n. Male genitalia: **A** lateral **B** segment IX and tergum X, dorsal **C** inferior appendage, ventral **D** inferior appendage, dorsal **E** inferior appendages, oblique lateral **F** phallic apparatus, lateral **G** apex of endotheca, ventral.

##### Paratypes.

**COLOMBIA:** **Valle:** same data as holotype, 12 males 17 females (pinned) (UMSP) (NMNH); Municipio de Buenaventura, Río Escalerete, 1 km E casa de “Acua Valle", ca. 16 km SE Cordoba, 3°49'38"N, 76°52'15"W, 210 m, 2.xii.1997, F. Muñoz-Q. et al., 9 males, 4 females (pinned) (UMSP).

##### Etymology.

The species name should be considered an arbitrary combination of letters, suggested by its similarity in form to that of other species in this species group (i.e., *Chimarra emima* Ross, 1959; *Chimarra onima* Flint, 1991; and *Chimarra jemima* Blahnik & Holzenthal, 1992).

### *Chimarra (Chimarra) poolei* group

Blahnik (1998) recognized 10 species in this group, broadly distributed from Costa Rica in Central America through the Brazilian subregion of South America, including southeastern Brazil. Species are readily recognized by the spine-like projections from the posterodorsal margin of segment IX (as distinguished from the spine-like modifications of tergum X in several species of the *Chimarra picea* group). All of the species have very short inferior appendages, either without or with very small dorsomesal projections, not generally evident in lateral view. The group is closely related to the *Chimarra bidentata* group. The 2 new species recognized here increase the species group to 12 species. The new species and new records of this group from Bolivia also represent a range extension for the species group.

#### Chimarra (Chimarra) cauca

Blahnik & Holzenthal sp. n.

urn:lsid:zoobank.org:act:5E55AA28-BEDD-4D2B-80EF-6BC8B90C74E8

http://species-id.net/wiki/Chimarra_cauca

Figs 6A–F 13

##### Description. 

This new species is most closely related to *Chimarra zamora* Blahnik, 1998. Notable synapomorphies are found in the overall structure of the lateral lobes of tergum X, which in both species have a somewhat concavely developed apical protuberance and also projecting, sensilla-bearing lateral projections. In *Chimarra cauca*, the sensilla-bearing projections are larger and more broadly rounded. Additionally, the spine-like processes of segment IX are much more prominent in *Chimarra cauca*. Female genitalia for the 2 species, while having some similarities, are also different, confirming that these are indeed distinct species.

*Adult*. Forewing length (male) 4.8–5.4 mm, (female) 5.7 mm. Cuticle of head and thorax dark brown, setae of anteromesal and frontal setal warts light brown, setae of other setal warts dark brown, grizzled (grayish in part or intermixed), femora brown, otherwise color nearly uniformly brownish-black (fuscous), including appendages and antennae. Postocular parietal sclerite short (not extended behind eye). Second segment of maxillary palp much shorter than segment 3 (about 2/3 length). Male protarsal claws enlarged, asymmetrical in size and shape, outer claw larger, twisted, nearly linear apically.

*Male genitalia*. Abdominal segment IX, in lateral view, with pronounced linear extension of anteroventral margin and with distinct small apodemes from anterodorsal margin; posterodorsal margin with prominent pair of acute, spine-like projections, extending about 2/3 length of tergum X; posteroventral process prominent, subtriangular, broad basally, acute apically, length slightly greater than width at base. Tergum X with mesal lobe membranous; lateral lobes sclerotized, moderately elongate, each with distinct, preapical, lateral projection, more or less concave on anterior margin (acute as viewed dorsally), lobe laterally with rounded projection bearing 2 sensilla. Preanal appendage short, knob-like. Inferior appendage, in lateral view, very short and strongly cupped basally; apical margin truncate, dorsally with very short, mesally curved process, process very angularly bent. Phallotheca with acute apicoventral projection; endotheca membranous, apparently with numerous minute spines and cluster of short sclerotized spines, endothecal spines 2, basal one moderately elongate, apical one short. Phallotremal sclerite complex composed of rod and ring structure, rod relatively short and ring with small, but distinct apicodorsal extension; complex also with small lateral sclerites.

*Female genitalia*. Sternum IX with ventral lobes short, subacute apically, laterally with small rounded clasper receptacles. Vaginal apparatus distinctly sclerotized apically, with sclerotized mesal lobe extending less than ½ length of vaginal apparatus, lobe with distinctly sclerotized lateral margins, appearing as sclerotized ridges; preapically with conspicuous, sclerotized, rounded lateral lobes; vaginal apparatus laterally with small rounded, lightly sclerotized projections at about midlength, each with acute awl-like apical projection; anterior half of vaginal apparatus membranous, parallel-sided, anteriorly with deflexed cup-like sclerite.

**Figure 6.**
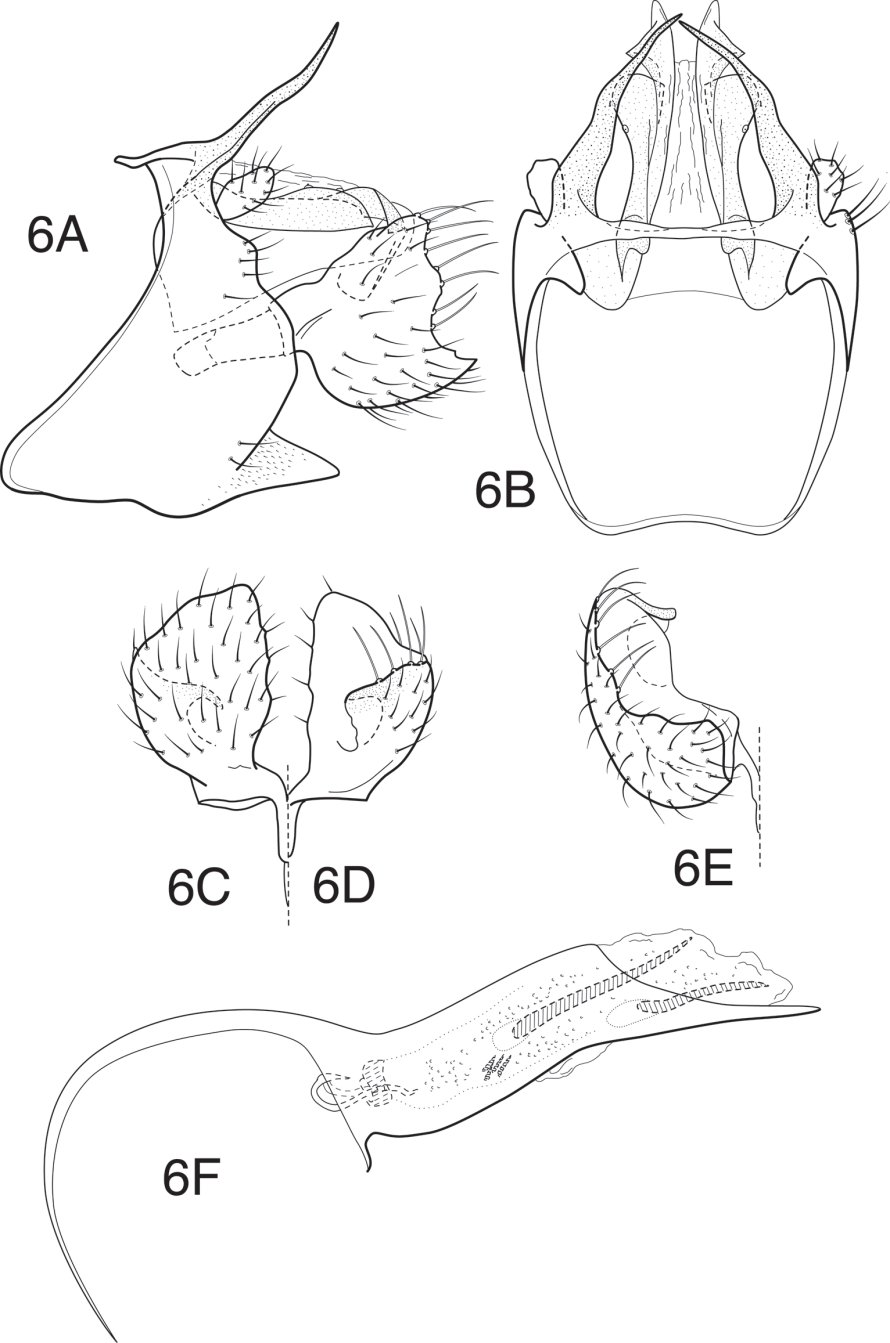
*Chimarra (Chimarra) cauca* sp. n. Male genitalia: **A** lateral **B** segment IX and tergum X, dorsal **C** inferior appendage, ventral **D** inferior appendage, dorsal **E** inferior appendage, caudal **F** phallic apparatus, lateral.

##### Holotype

**, male** (pinned) (UMSP000108722)**:** **COLOMBIA:** **Cauca:** Municipio de Inzá, Quebrada San Andrés, ca. 500 m, W Restaurante “La Portada”, San Andrés de Pisimbalá, 2°34'56"N, 76°2'36"W, 1750 m, 21.xii.1997, F. Muñoz-Q. et al. (UMSP).

##### Paratypes.

**COLOMBIA:** **Cauca:** same data as holotype, 4 males (pinned) (UMSP) (NMNH); Municipio de Belalcazar, Quebrada Tálaga, ca. 14 km N Páez (Belalcazar), 2°42'24"N, 76°1'5"W, 1680 m, 22.xii.1997, F. Muñoz-Q. et al., 2 males (pinned) (UMSP); **Magdalena:** Municipio Ciénaga, ca. 25 km NW Estación Exp. San Lorenzo, Sierra Nevada de Santa Marta, Río Cordoba, 11°2'22"N, 74°2'18"W, 930 m, 12.xii.1997, F. Muñoz-Q. et al., 1 male, 1 female (pinned) (UMSP).

##### Etymology.

This species is named *Chimarra cauca*, used as noun in apposition, for the Department (regional area) in Colombia where the type specimens were collected.

#### Chimarra (Chimarra) desirae

Blahnik & Holzenthal sp. n.

urn:lsid:zoobank.org:act:AD05D344-F0D0-40FA-B7A8-76EA9E98E4C6

http://species-id.net/wiki/Chimarra_desirae

Fig. 7A–E

##### Description. 

This is a distinctive species, most closely related to *Chimarra adamsae* Blahnik, 1998. The most distinctive diagnostic feature, as in most species of the *Chimarra poolei* group, is found in the structure of the lateral lobes of tergum X. In *Chimarra desirae* these are distinctly sclerotized and have an upright basal projection, and a dorsoventrally flattened lateral crease, so that the apices of the lobes, in dorsal view, appear broadly rounded. *Chimarra adamsae* has these same general features, but the upright processes are subquadrate and more apical, and the lateral creases are also more apical, resulting in the apices being narrow, as viewed either laterally or dorsally. Additionally, the sensilla of the lateral lobes in *Chimarra desirae* are located along the lateral crease, whereas those of *Chimarra adamsae* are basal to the crease.

*Adult*. Forewing length (male) 4.2–5.0 mm. Cuticle of head and thorax very dark, nearly black, setae of anteromesal and frontal setal warts whitish, setae of other setal warts brownish-black, grizzled (grayish in part or intermixed), otherwise color nearly uniformly brownish-black (fuscous), including appendages and antennae. Postocular parietal sclerite short (not greatly extended behind eye). Second segment of maxillary palp much shorter than segment 3 (about 2/3 length). Male protarsal claws enlarged, asymmetrical in size and shape, outer claw much larger, twisted, nearly linear apically.

*Male genitalia*. Abdominal segment IX, in lateral view, with anteroventral margin sinuously extended from dorsal margin; anterodorsal margin with short apodemes; posterodorsal margin with pair of acute, spine-like projections, extending about 1/3 length of tergum X; posteroventral process subtriangular, relatively broad basally, subacute apically. Tergum X with very short membranous mesal lobe and 2 sclerotized lateral lobes, each bearing large, upright, broadly rounded basal lobe; lateral lobe also with strongly dorsoventrally flattened lateral crease, extending from near base to apex, base of crease with 2 slightly raised sensilla; terminus of lateral lobe narrowed and slightly declivous as viewed laterally, broadly rounded as viewed dorsally. Preanal appendage short, knob-like. Inferior appendage, in lateral view, very short and strongly cupped basally; dorsomesally with small, barely suggested, angular process. Phallotheca with distinct, elongate, apicoventral extension; ventral surface of phallotheca with lightly sclerotized projection; dorsal surface of endotheca with preapical tract of minute spines, basal ones slightly longer, endothecal spines 2, basal one moderately elongate, apical one short and curved. Phallotremal sclerite complex composed of rod and ring structure, rod relatively short and ring with small, but distinct, apicodorsal extension; rod preapically with 2 small, curved, symmetric sclerites.

*Female genitalia*. Female unknown.

**Figure 7.**
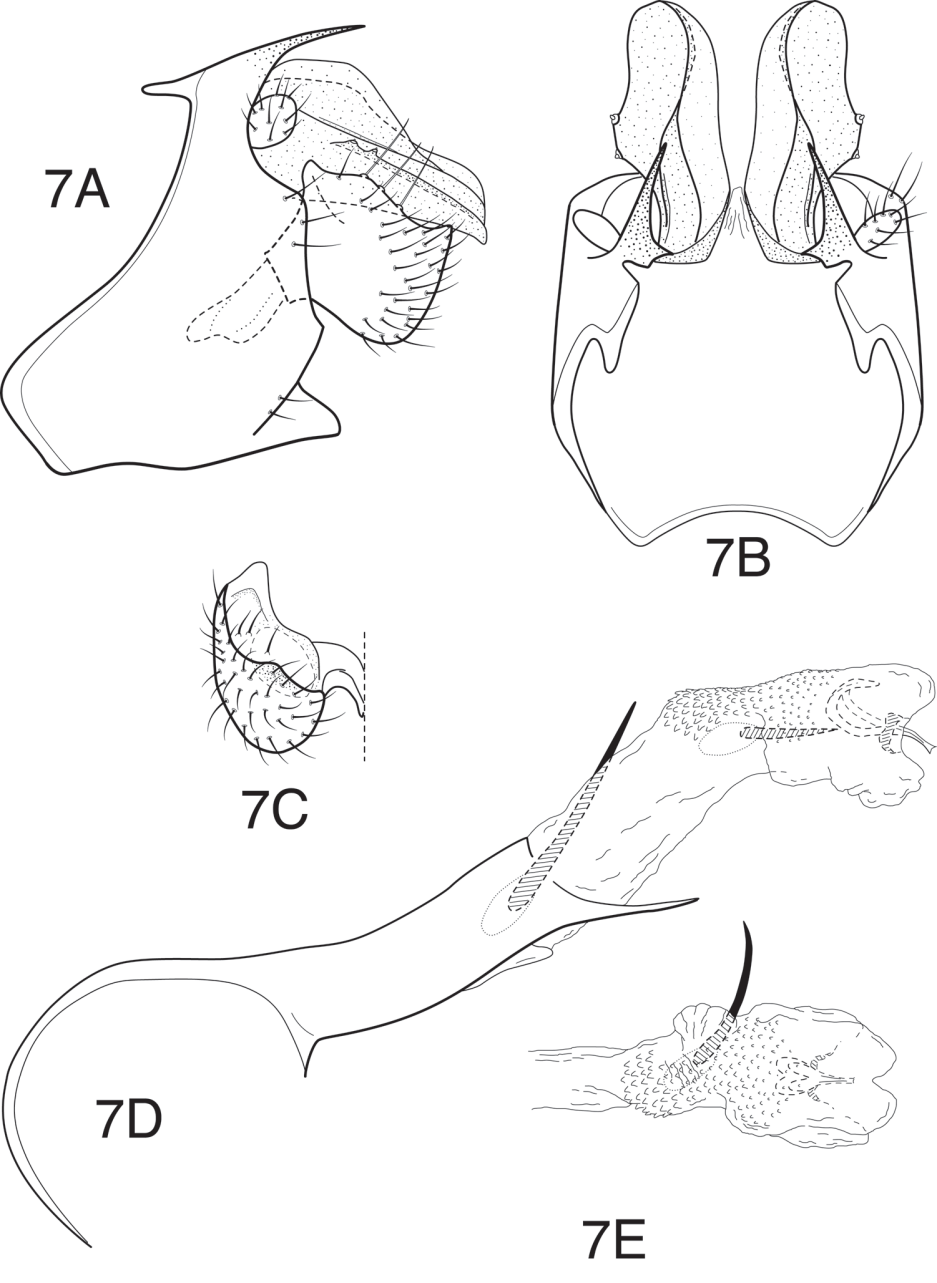
*Chimarra (Chimarra) desirae* sp. n. Male genitalia: **A** lateral **B** segment IX and tergum X, dorsal **C** inferior appendage, caudal **D** phallic apparatus, lateral **E** apex of endotheca, dorsal.

**Figures 8–13.**
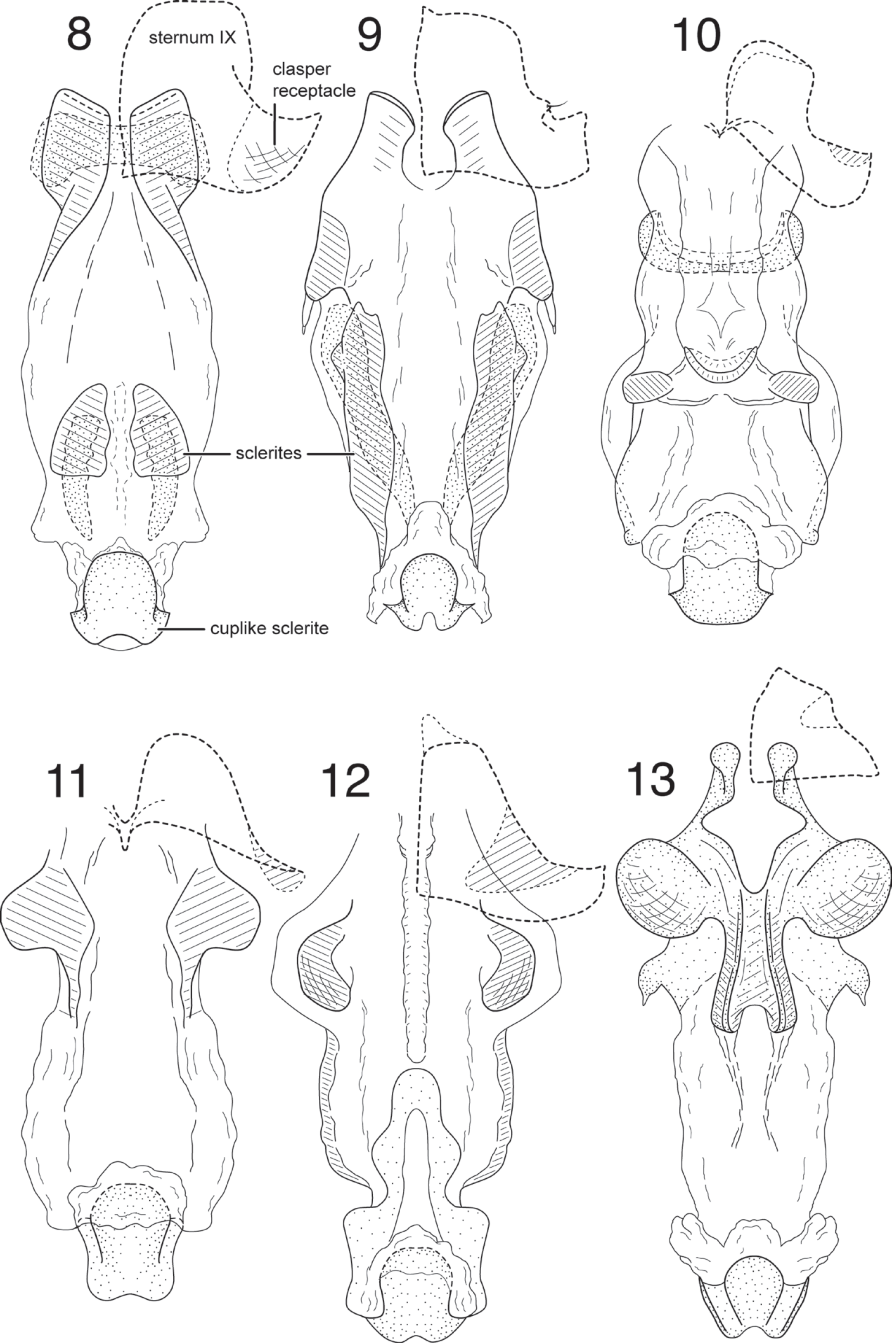
*Chimarra (Chimarra)* female genitalia, ventral view of vaginal apparatus: **8** *Chimarra calori* sp. n. **9** *Chimarra onchyrhina* sp. n. **10** *Chimarra inchoata* sp. n. **11** *Chimarra nicehuh* sp. n. **12** *Chimarra sunima* sp. n. **13** *Chimarra cauca* sp. n.

##### Holotype

**, male** (pinned) (UMSP000131058)**:** **BOLIVIA:** **La Paz:** AMNI Madidi, San Migual de Bala, Arroyo Bacuatra Grande, 14°30.737'S, 67°31.385'W, 280 m, 17–19.vii.2003, Robertson, Blahnik, Apaza (UASC).

##### Paratypes.

**BOLIVIA:** **La Paz:** same data as holotype, 8 males (pinned) (UMSP) (NMNH).

##### Etymology.

We take great pleasure in naming this species *Chimarra desirae* for Dr. Desiree Robertson-Thompson, who collected the type specimens, in acknowledgment of her contributions to the study of Neotropical caddisflies.

### *Chimarra (Chimarrita) simpliciforma* group

Blahnik (1997) recognized 10 species in this species group, which represents the largest and most broadly distributed of the 3 species groups recognized for the subgenus. Described species are mostly known from very few specimens. Species have been recorded from the Guyana Highlands, Amazon Basin, and southeastern Brazil, thus spanning a very broad area. The apparent disjunct distribution of the group probably reflects a very inadequate knowledge about the actual distribution of known species and also the species diversity of the group. Currently 4 species are known from southeastern Brazil. The 2 new species described here are closely related to 2 of the described species. Species in the group are easily recognized by structure of the phallic apparatus in the males, which has a single and typically elongate spine that emerges from the dorsum of the phallotheca. Females in the subgenus are also distinctive because of their elongate genitalia.

#### Chimarra (Chimarrita) curvipenis

Blahnik & Holzenthal sp. n.

urn:lsid:zoobank.org:act:67D04EC4-7571-4CA9-9513-93D906CD4C20

http://species-id.net/wiki/Chimarra_curvipenis

Fig. 14A–F 16A–B

##### Description. 

*Chimarra curvipenis*, sp. n. is similar to *Chimarra kontilos* Blahnik, 1997 and represents a closely related sister species. Similarities are found in the general structure of the inferior appendages and tergum X of the male. However, there are a number of differences. Tergum X has a small lateral, sensilla-bearing projection at past midlength and the inferior appendages are more elongate and have more acute apices. Additionally, the phallotheca is quite different, distinctly curved, rather than elongate, tubular, and the apex of the dorsal phallic spine lacks the distinctive whip-like extension found in *Chimarra kontilos*. In the key by Blahnik (1997), *Chimarra curvipenis* would come out with *Chimarra tortuosa* Blahnik, from which it differs significantly in the shape of the inferior appendages (longer and more strongly incurved apically), and structure of the phallic apparatus (phallotheca more strongly curved and phallic spine less sinuously curved).

*Adult*. Forewing length (male 4.8 mm, (female) 4.8 mm. Overall color nearly uniformly light brown, palps slightly darker; thorax ventrally, meso- and metacoxae golden brown. Venational branching of forewing typical for *Chimarra*; Rs straight *s*, *r* and *r-m* of forewing nearly linearly arranged and unpigmented, as is *m-cu* and apex of Cu_2_; 2A of forewing with apparent apical “fork”, that to 1A elongate and broadly rounded, that to 2A very short (appearing as cross-vein). Rs of hind wing 4-branched, M 3-branched. Head short (postocular parietal sclerite short). Maxillary palps relatively short, segment 2 longer than 3. Male with protarsi unmodified.

*Male genitalia*. Segment IX relatively wide; as viewed laterally, with anterior margin concave, posterior margin angularly projecting at level of inferior appendages; anteroventral margin noticeably expanded, apex of expansion acutely rounded (as viewed dorsally or ventrally); ventral process narrow, elongate, acute, somewhat curved. Tergum X moderately elongate, fused to segment IX, apex with deep, U-shaped mesal excision, extending about 1/3 length of tergum, forming narrow, paired lobes apically (as viewed dorsally); tergum laterally with short, rounded projections at just past midlength (basal to apical lobes); apical lobes and lateral projections with numerous sensilla. Preanal appendage very small, rounded, fused near base of tergum X. Inferior appendage relatively elongate, nearly linear, except apex deflexed, narrowed, and mesally curved, apex forming acute projection. Phallotheca with somewhat bulbous base, otherwise elongate, narrow, ventral margin projecting and strongly curved; phallic spine single, stout, curved, very elongate (subequal in length to phallotheca), emerging dorsally near base of phallotheca and with slight sinuous twist. Phallotremal sclerite complex (if present) indistinct.

*Female genitalia*—Sternum VII with ventral process; process projecting, subacute, located near middle of segment, as viewed laterally. Segment VIII synsclerous, short dorsally, anterolateral margin nearly straight, indented and narrowed dorsally, rounded ventrally; segment sclerously connected ventrally to sternum IX; anteroventral margin of segment, as viewed ventrally, with deep, mesal emargination extending almost entire length of segment, emargination strongly narrowed posteriorly, bordered laterally by distinct U-shaped sclerotization. Sternum IX elongate, with paired, angular projections, projections continuous posteriorly with elongate, narrow ventral sclerites; sternum membranous ventrally between paired sclerites, and membranous also laterally from acute basal projection to apex. Tergum IX elongate, narrow, slightly curved, moderately setose, anteroventrally with short apodemes. Segment X with elongate basal portion, furrowed dorsally, with mesal tract of setae in furrow; apically with pair of small, rounded, setose lobes, each with short apical cercus. Vaginal apparatus largely membranous, with indistinct sclerites, anterior one forming narrow ring.

**Figure 14.**
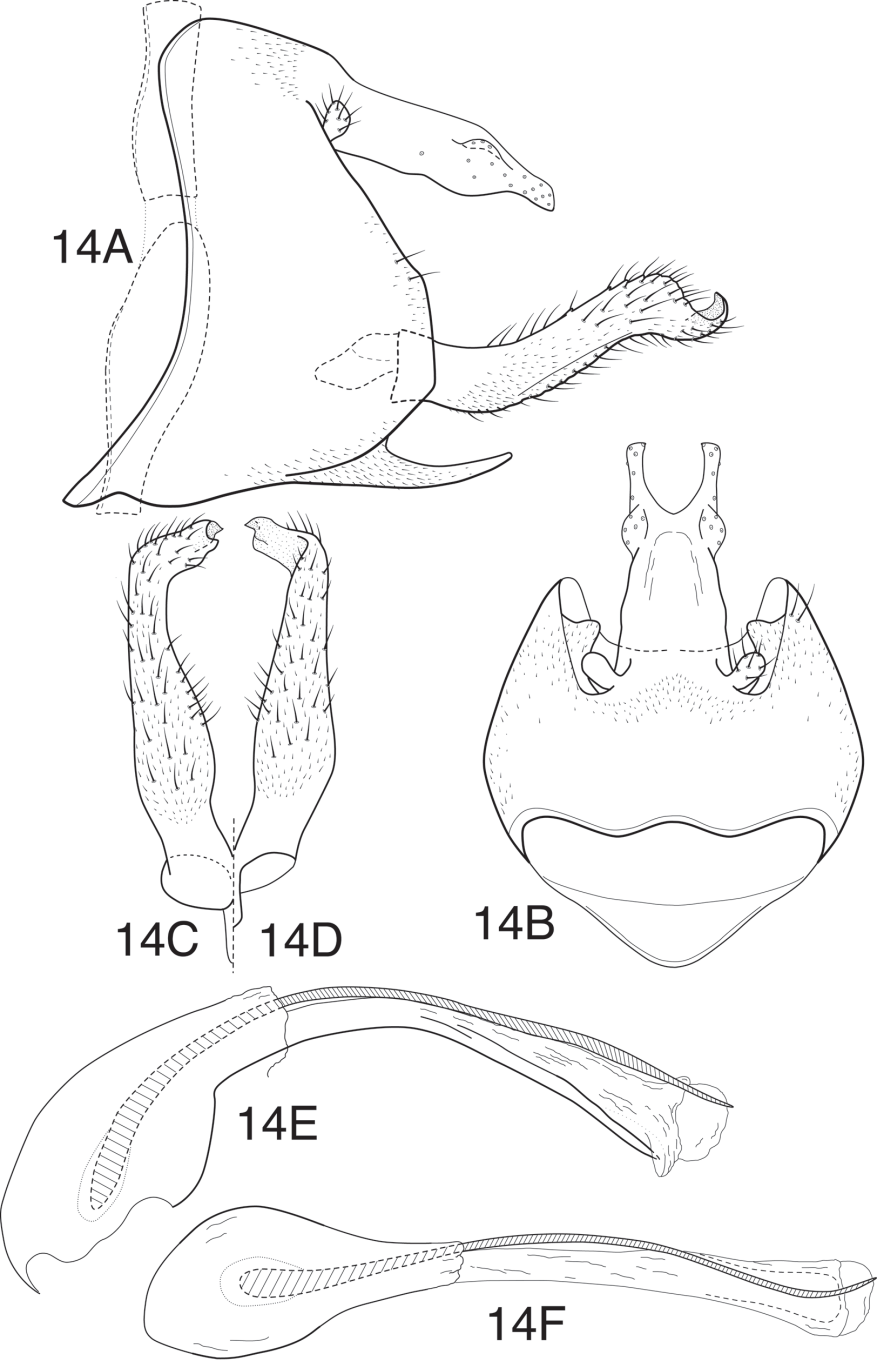
*Chimarra (Chimarrita) curvipenis* sp. n. Male genitalia: **A** lateral **B** segment IX and tergum X, dorsal **C** inferior appendage, ventral **D** inferior appendage, dorsal **E** phallic apparatus, lateral **F** phallic apparatus, dorsal.

##### Holotype

**, male** (pinned) (UMSP000033831)**:** **BRAZIL:** **Minas Gerais:** Serra do Cipó, Capão da Mata, 19°19.347'S, 43°32.249'W, 1170 m, 13–14.ii.1998, Holzenthal & Paprocki (MZUSP).

##### Paratypes.

**BRAZIL:** **Minas Gerais:** same data as holotype, 1 female (pinned) (UMSP).

##### Etymology.

This species is named *Chimarra curvipenis* for its curved or bent phallotheca, a character that helps to distinguish it from *Chimarra kontilos*.

#### Chimarra (Chimarrita) latiforceps

Blahnik & Holzenthal sp. n.

urn:lsid:zoobank.org:act:E5B89261-77C0-4223-AA64-D3D296062339

http://species-id.net/wiki/Chimarra_latiforceps

Figs 15A–E 17A–B

##### Description. 

*Chimarra latiforceps* is very similar to *Chimarra majuscula* Blahnik, 1997, particularly in the general shape of the inferior appendages and tergum X of the male, and would key out to that species in the key by Blahnik (1997). However, it does differ in details of both structures. It is also somewhat darker in overall color. The most diagnostic difference is in the shape of the inferior appendages, which are broader overall and have more truncate apices. Tergum X differs in that the lateral lobes formed by the mesal invagination are narrow, elongate, rather than inflated apically.

*Adult*. Forewing length (male 4.8–6.0 mm, (female) 5.2–5.9 mm. Overall color nearly uniformly medium brown, palps slightly darker; thorax ventrally, meso- and metacoxae light brown, antennae indistinctly annulated. Venational branching of forewing typical for *Chimarra*; Rs straight *s*, *r* and *r-m* of forewing nearly linearly arranged and unpigmented, as is *m-cu* and apex of Cu_2_; 2A of forewing with apparent apical “fork”, that to 1A elongate and broadly rounded, that to 2A very short (appearing as cross-vein). Rs of hind wing 4-branched, M 3-branched. Head very short and somewhat flattened (postocular parietal sclerite short). Maxillary palps relatively short, segment 2 slightly longer than 3. Male with protarsi unmodified.

*Male genitalia*. Segment IX relatively wide; as viewed laterally, with anterior margin concave, posterior margin very distinctly, angularly projecting at level of inferior appendages; anteroventral margin expanded, apex of expansion acutely rounded mesally (as viewed dorsally or ventrally); ventral process narrow, elongate, acute, somewhat curved. Tergum X relatively short (much shorter than inferior appendages), fused to segment IX, apex with deep, U-shaped mesal excision, extending about ½ length of tergum, forming narrow, paired lobes apically (as viewed dorsally); apical lobes and lateral margins of tergum with numerous sensilla. Preanal appendage very small, rounded, fused near base of tergum X. Inferior appendage relatively elongate, nearly linear, apex incurved and broadly subtruncate. Phallotheca with somewhat bulbous base, otherwise moderately elongate, narrow, distinctly curved; phallic spine single, stout, curved, elongate (subequal in length to phallotheca), emerging dorsally near base of phallotheca; endotheca moderately elongate, slightly inflated in basal ½. Phallotremal sclerite complex evident as elongate, narrow, sclerotized rod.

*Female genitalia*. Sternum VII with ventral process; process large, projecting, subacute, emerging near anterior margin of segment, as viewed laterally. Segment VIII synsclerous, short dorsally, anterolateral margin broadly rounded, indented and narrowed dorsally, rounded ventrally; segment sclerously connected ventrally to sternum IX; anteroventral margin of segment, as viewed ventrally, with short, narrow mesal emargination, margins of emargination distinctly sclerotized, sclerotization extending posteriorly as pair of distinct ridges. Sternum IX elongate, with paired, angular projections, projections continuous posteriorly with elongate, narrow ventral sclerites; sternum membranous ventrally between paired sclerites, and membranous also laterally from acute basal projection to apex. Tergum IX elongate, narrow, slightly curved, moderately setose, anteroventrally with short apodemes. Segment X with elongate basal portion, furrowed dorsally, with mesal tract of setae in furrow; apically with pair of small, rounded, setose lobes, each with short apical cercus. Vaginal apparatus largely membranous, with indistinct sclerites, anterior one forming narrow ring.

**Figure 15.**
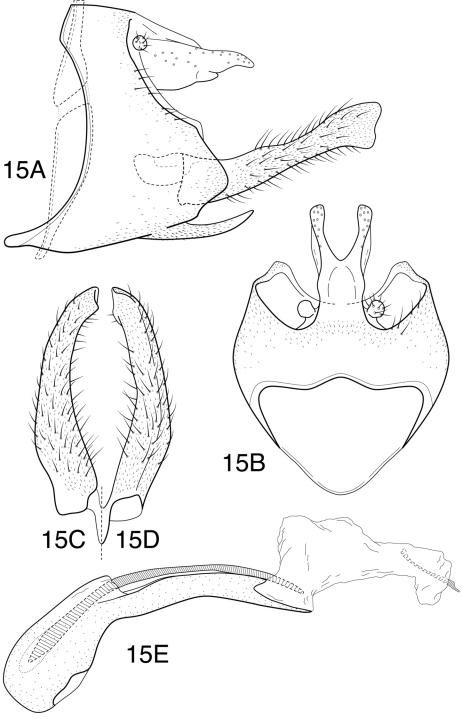
*Chimarra (Chimarrita) latiforceps* sp. n. Male genitalia: **A** lateral **B** segment IX and tergum X, dorsal **C** inferior appendage, ventral **D** inferior appendage, dorsal **E** phallic apparatus, lateral.

**Figures 16–18.**
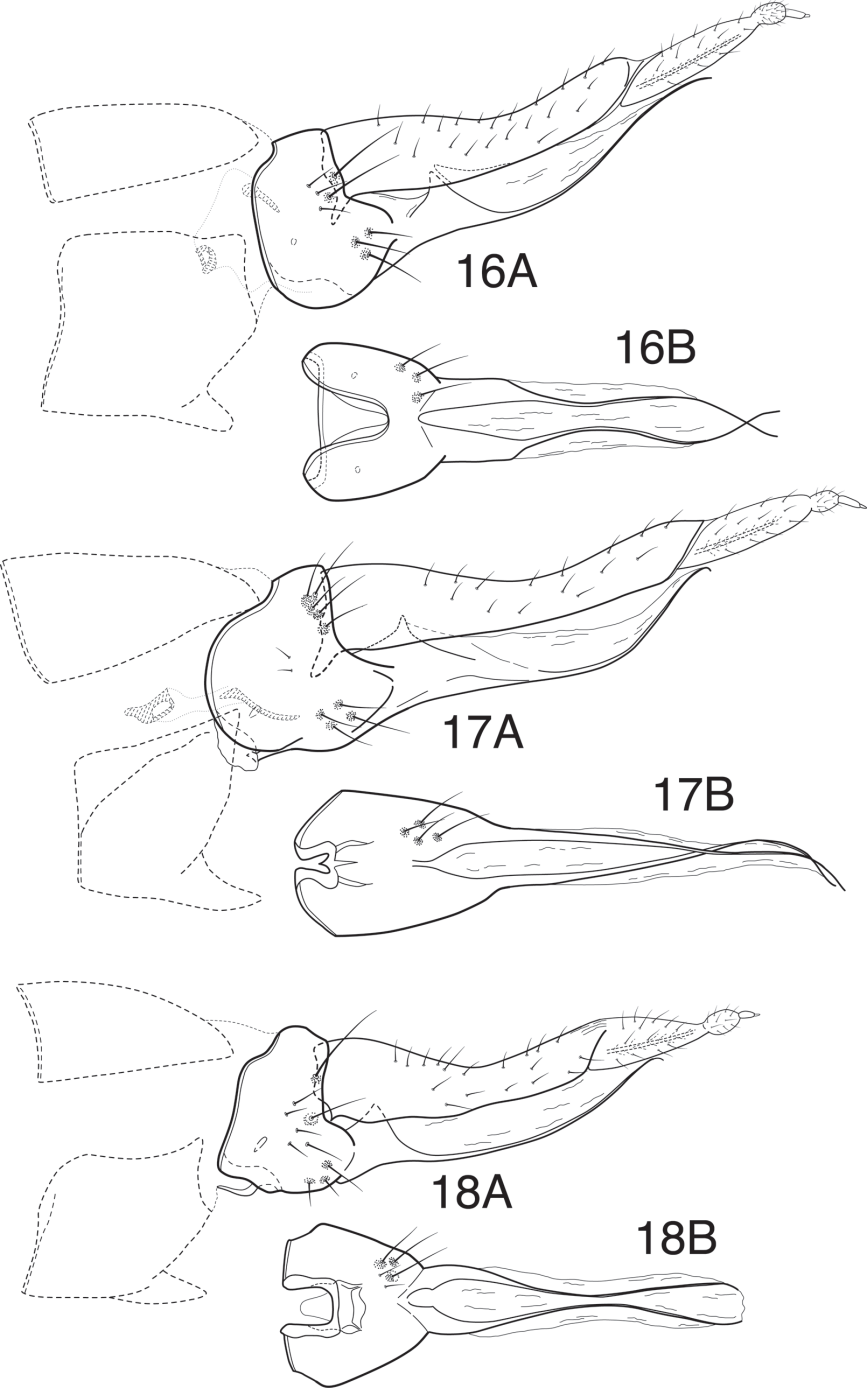
*Chimarra (Chimarrita)* female genitalia: **16** *Chimarra curvipenis* sp. n.: **A** lateral **B** ventral **17** *Chimarra latiforceps* sp. n.: **A** lateral **B** ventral **18** *Chimarra camella* Blahnik: **A** lateral **B** ventral.

##### Holotype

**, male** (pinned) (UMSP000200929)**:** **BRAZIL:** **São Paulo:** Parque Estadual de Campos do Jordão, Rio Galharda, 22°41.662'S, 45°27.783'W, 1530 m, 13–15.ix.2002, Blahnik, Prather, Melo, Huamantinco (MZUSP).

##### Paratypes.

**BRAZIL:** **Minas Gerais:** Crucilándia, Fazenda Nova Limeira, 20°23.131'S, 44°22.071'W, 875 m, 22–23.ii.1998, Holzenthal, Paprocki, Huisman, 5 males, 1 female (pinned) (UMSP); Parque Estadual do Itacolomi, trib. to Rio Belchior, 20°25.302'S, 43°25.697'W, 700 m, 6.xi.2001, Holzenthal, Paprocki, Blahnik, Amarante, 1 male, 1 female (pinned) (NMNH); Parque Estadual do Ibitipoca, spring trib. near director's house, 21°42.695'S, 43°53.760'W, 1357 m, 19–20.xi.2001, Paprocki & Blahnik, 3 males, 7 females (alcohol) (UMSP); **São Paulo:** Estação Biológica Boraceia, Rio Coruja, 23°40.10'S, 45°53.95'W, 850 m, 18.iv.1998, Holzenthal, Melo, Froehlich, 1 male, 1 female (pinned) (UMSP); Estação Biológica Boraceia, Rio Venerando & tribs., 23°39.185'S, 45°53.414'W, 850 m, 18–20.iv.1998, Holzenthal, Melo, Froehlich, 1 male (pinned) (UMSP); Rio Casquilho, 3.4 km NE Parque Estadual Campos do Jordão, 22°40.29'S, 45°27.87'W, 1550 m, 23.i.1998, Holzenthal, Froehlich, Paprocki, 1 male (pinned) (MZUSP); same data as holotype except, 22.i.1998, Holzenthal, Froehlich, Paprocki, 1 male (pinned) (UMSP); Parque Nacional da Serra da Bocaina, Cachoeira Santo Izidro, 22°44.830'S, 44°36.882'W, 1480 m, 2.iii.2002, Blahnik & Paprocki, 1 male (pinned) (MZUSP).

##### Etymology.

This species is named *Chimarra latiforceps*, from the Latin words *latus*, wide, and *forceps*, a pincers or tong, in reference to the inferior appendages, which are shaped something like a pair of broad tipped forceps.

#### Chimarra (Chimarrita) camella

Blahnik

http://species-id.net/wiki/Chimarra_camella

Fig. 18A–B

##### Description. 

The female of this species was not previously illustrated. It is included here, since it is the only species of the subgenus for which the female is not illustrated. The female can be distinguished from other species by the nearly linear anterior margin of segment VIII and, especially, by the distinctive, subquadrate invagination of the posteroventral margin of the same segment. The mesal margin of the invagination has a lightly sclerotized, tab-like projection.

*Female genitalia*. Sternum VII with ventral process; process projecting, subacute, located near middle of segment, as viewed laterally. Segment VIII synsclerous, short dorsally, anterolateral margin nearly straight, indented and narrowed dorsally and ventrally; segment sclerously connected ventrally to sternum IX; anteroventral margin of segment, as viewed ventrally, with subquadrate mesal emargination, extending about ½ length of segment, margins of emargination very distinctly sclerotized, sclerotization extending somewhat posteriorly, mesal margin of emargination with lightly sclerotized tab-like projection. Sternum IX elongate, with paired, angular projections, projections continuous posteriorly with elongate, narrow ventral sclerites; sternum membranous ventrally between paired sclerites, and membranous also laterally from acute basal projection to apex. Tergum IX elongate, narrow, distinctly curved, moderately setose, anteroventrally with apodemes only suggestively developed. Segment X with elongate basal portion, furrowed dorsally, with mesal tract of setae in furrow; apically with pair of small, rounded, setose lobes, each with short apical cercus. Vaginal apparatus largely membranous, sclerites (if present) indistinct.

##### Material examined.

**BRAZIL: Minas Gerais:** Parque Estadual do Rio Preto, trib. to Rio Preto, 18°06.879'S, 43°20.595'W, 700 m, 20.v.1998, Holzenthal & Paprocki, 1 male, 1 female (UMSP); Parque Estadual do Rio Preto, Rio Preto, 18°06.993'S, 43°20.373'W, 650 m, 19.v.1998, Holzenthal & Paprocki, 1 male (UMSP); Parque Nacional do Caparaó, Rio Caparaó at Vale Verde, 20°25.029'S, 41°50.767'W, 1350 m, 12–13.iii.2002, Holzenthal, Blahnik, Paprocki, Pather, 1 male (UMSP); Serra do Cipó, Capão da Mata, 19°19.347'S, 43°32.249'W, 1170 m, 10.iii.1996, Holzenthal, Rochetti, Oliveira, 1 female (UMSP); **Rio de Janeiro:** Parque Nacional do Itatiaia, Rio Campo Belo, trail to Veu da Noiva, 22°25.706'S, 44°37.171'W, 1310 m, 5.iii.2002, Holzenthal, Blahnik, Paprocki, Prather, 1 male (UMSP); **São Paulo:** Estação Biológica Boraceia, Rio Venerando 7 tribs. 23°39.185'S, 45°53.414'W, 850 m, 18–20.iv.1998, Holzenthal, Melo, Froehlich, 4 males, 6 females (UMSP); Parque Nacional da Serra da Bocaina, Cachoeira das Posses, 22°46.437'S, 44°36.250'W, 1250 m, 3.iii.2002, Blahnik, Paprocki, Prather, 1 male, 2 females (MZUSP); Estação Biológica Boraceia, Rio Coruja at bridge, 23°40.10'S, 45°53.95'W, 850 m, 20.ix.2002, Blahnik, Melo, Froehlich, Silva, 1 male (UMSP).

### Chimarra (Otarrha)

The subgenus *Otarrha* was established by Blahnik (2002) to include 31 species, broadly distributed in northern South America and in the Greater and Lesser Antilles, with individual species also found in lower Central America and southeastern Brazil. A clade of 10 described species occurs in the Greater Antilles. All of these were treated in the work by Blahnik (2002), except for *Chimarra koki* Botosaneanu (1996), described from the Dominican Republic, and a subsequently described subspecies of *Chimarra spinulifera* Flint, 1968, *Chimarra spinulifera baoruco* Flint & Sykora, 2004, also from the Dominican Republic. The new species described here increases the number of species in the subgenus known from Cuba to 5, and the number of species known from the Greater Antilles to 11.

#### Chimarra (Otarrha) soroa

Blahnik & Holzenthal sp. n.

urn:lsid:zoobank.org:act:7D1E3A56-5233-43BD-9B2A-D9C49211CC40

http://species-id.net/wiki/Chimarra_soroa

Fig. 19A–F 20A–C

##### Description. 

This new species closely resembles *Chimarra garciai* Botosaneanu, 1980, also known from Cuba, and represents a probable sister species. It is also similar in general features to *Chimarra jamaicensis* Flint, 1968 and *Chimarra machaerophora* Flint, 1968, both described from Jamaica. All of these species have a tergum X that is divided mesally, with very elongate lateral lobes, and similarly shaped inferior appendages. Like *Chimarra garciai*, *Chimarra soroa*, sp. n. has a tergum VIII with a pair of short spine-like projections, but without the mesal projection of *Chimarra machaerophora*. In the key by Blahnik (2002), *Chimarra soroa* would key out to the couplet including *Chimarra garciai* and *Chimarra jamaicensis*. It differs from *Chimarra garciai* in lacking a pair of sclerotized spines at the base of the lobes of tergum X, and from either species in having a distinctly projecting, rounded, sensilla-bearing process on the lateral margin of each of the lobes of tergum X. Additionally, the inferior appendages appear more truncate in lateral view than in either of those species.

*Adult*. Forewing length (male) 4.8–5.2 mm, (female) 5.3 mm. Overall color nearly uniformly medium brown, palps slightly darker; thorax ventrally, meso- and metacoxae light brown. Venation typical for subgenus, forewing with Rs straight *s*, *r* and *r-m* of forewing linearly arranged and unpigmented, as is *m-cu* and apex of Cu_2_; 2A of forewing without apical “fork” (2A and 3A both apparently looped to 1A). Rs of hind wing 3-branched, M 2-branched. Head relatively short (postocular parietal sclerite short). Maxillary palps short, segment 2 much shorter than 3 (about ½ length). Male with protarsi unmodified.

*Male genitalia*. Tergum VIII with short, paired, acute processes from posterior margin. Abdominal segment IX widest mid-laterally, anterolateral margin broadly rounded, narrowed dorsally and ventrally, posterolateral margin angularly projecting at level of inferior appendages; anterodorsal and anteroventral margins strongly invaginated mesally; posteroventral process elongate, projecting, narrowed basally, rounded apically. Tergum X divided mesally, forming 2 elongate sclerotized lateral lobes; each strongly tapered and somewhat arched, with scattered sensilla apically and more densely on projecting, rounded, basolateral projection. Preanal appendage very large, flattened, earlike. Inferior appendage short, truncate apically in lateral view, with short, broadly rounded process on mesal surface. Phallic apparatus with phallotheca narrow, tubular, expanded basodorsally; endotheca membranous, apparently moderately elongate (incompletely expanded in holotype). Phallotremal sclerite complex composed of rod and ring structure, about ¼ length of phallotheca; ring distinct, with dorsal projection, rod only relatively elongate, with small apicolateral sclerites.

*Female genitalia*. Segment VIII short; anterior margin slightly projecting midlaterally at position of apodemes, apodemes short, angular, undeveloped; posterior margin nearly straight, with deep cleft near ventral margin, extending almost to anterior margin, ventral part of segment (below cleft) forming projecting process, narrow as viewed laterally, rounded apically as viewed ventrally; segment with elongate setae on posterolateral margin and shorter setae at apex of ventral projection. Tergum IX very short, with moderately elongate apodemes from anteroventral margin; posteromesally with projecting, short, rounded, setose projection. Sternum IX membranous. Tergum X bilobed, each lobe divided into prominent, rounded, lightly sclerotized basal region and less sclerotized apical region with cercus at apex. Vaginal apparatus moderately elongate, dorsalaterally with elongate paired sclerites, each divided for nearly entire length to form apparent pair of rod-like sclerites; apically with additional pair of short needle-like sclerites on either side of elongate sclerites; vaginal apparatus anteriorly with indistinct ring-like sclerite.

**Figure 19.**
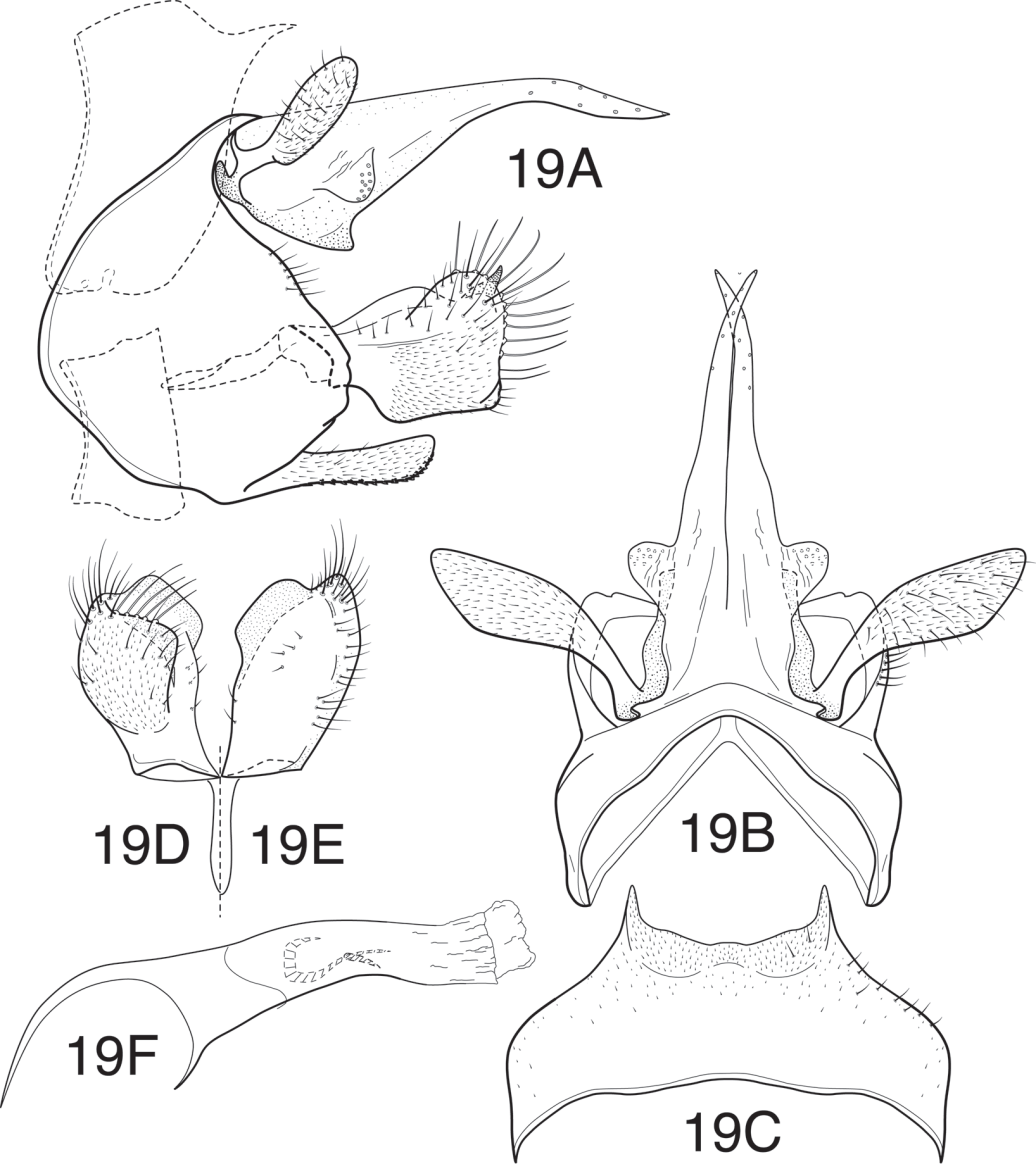
*Chimarra (Otarrha) soroa* sp. n. Male genitalia: **A** lateral **B** segment IX and tergum X, dorsal **C** tergum VIII, dorsal **D** inferior appendage, ventral **E** inferior appendage, dorsal **F** phallic apparatus, lateral.

**Figure 20.**
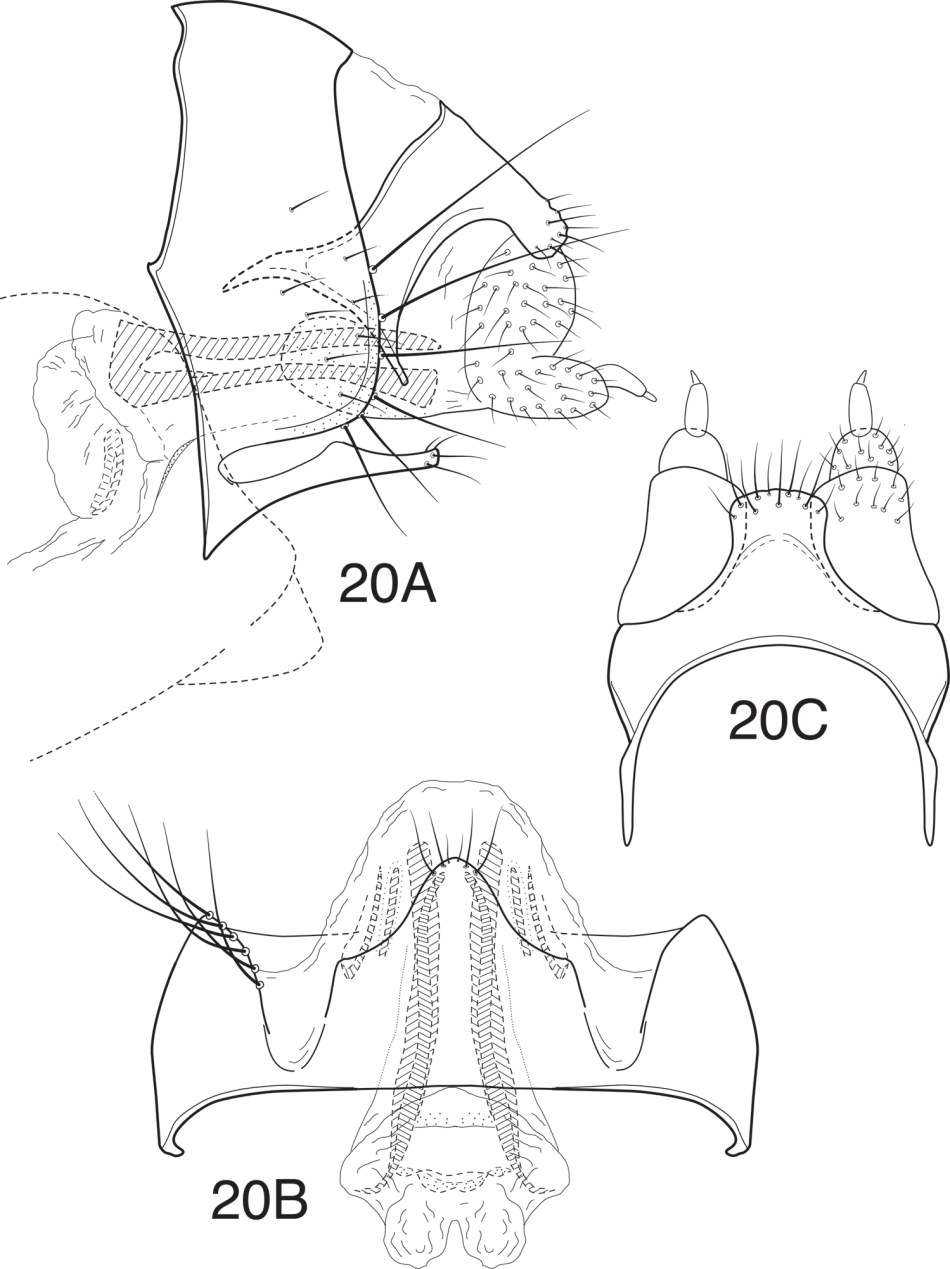
*Chimarra (Otarrha) soroa*, female genitalia: **A** lateral **B** ventral view of segment VIII and vaginal apparatus **C** dorsal of tergum IX and cerci.

##### Holotype

**, male** (pinned) (UMSP000095831)**:** **CUBA:** **Pinar del Río:** La Caridad, 2 km NW Soroa, 22°48.6'N, 83°01.2'W, 220 m, 4–5.xii.1994. O.S. Flint, Jr. (NMNH).

##### Paratypes.

**CUBA: Pinar del Río:** same data as holotype, 1 male (pinned) (NMNH); Soroa, 22°47.7'N, 83°00.1'W, 200 m, 4–6.xii.1994. O.S. Flint, Jr., 1 female (pinned) (NMNH).

##### Etymology.

This species is named *Chimarra soroa*, used as a noun in apposition, after the resort in Cuba near which the type specimen was collected.

## Supplementary Material

XML Treatment for Chimarra (Chimarra) calori

XML Treatment for Chimarra (Chimarra) onchyrhina

XML Treatment for Chimarra (Chimarra) inchoata

XML Treatment for Chimarra (Chimarra) nicehuh

XML Treatment for Chimarra (Chimarra) sunima

XML Treatment for Chimarra (Chimarra) cauca

XML Treatment for Chimarra (Chimarra) desirae

XML Treatment for Chimarra (Chimarrita) curvipenis

XML Treatment for Chimarra (Chimarrita) latiforceps

XML Treatment for Chimarra (Chimarrita) camella

XML Treatment for Chimarra (Otarrha) soroa

## References

[B1] Blahnik RJ (1997) Systematics of *Chimarrita*, a new subgenus of *Chimarra* (Trichoptera: Philopotamidae). Systematic Entomology 22: 199-243. doi: 10.1046/j.1365-3113.1997.d01-39.x

[B2] Blahnik RJ (1998) A revision of the Neotropical species of the genus *Chimarra*, subgenus *Chimarra* (Trichoptera: Philopotamidae). Memoirs of the American Entomological Institute 59: vi+1–318.

[B3] Blahnik RJ (2002) Systematics of *Otarrha*, a new Neotropical subgenus of *Chimarra* (Trichoptera: Philopotamidae). Systematic Entomology 27: 65-130. doi: 10.1046/j.0307-6970.2001.00166.x

[B4] Blahnik RJ Holzenthal RW (2004) Collection and curation of Trichoptera, with an emphasis on pinned material. Nectopsyche, Neotropical Trichoptera Newsletter 1: 8-20.

[B5] Blahnik RJ Holzenthal RW Prather AL (2007) The lactic acid method for clearing Trichoptera genitalia. In: Bueno-Soria J Barba-Álvarez R Armitage BJ (Eds). Proceedings of the 12th International Symposium on Trichoptera. The Caddis Press, Columbus, Ohio: 9-14.

[B6] Botosaneanu L (1996) Caddis flies (Trichoptera) from the Dominican Republic (West Indies). II. all families except Hydroptilidae; with general observations for Hispaniola. Bulletin de l’Institut Royal des Sciences Naturelles de Belgique, Entomologie 66: 5-26.

[B7] Bueno-Soria J Santiago-Fragoso S Barba-Alvarez R (2001) Studies in aquatic insects, XVIII: new species and new record of caddisflies (Trichoptera) from Mexico. Entomological News 112: 145-158.

[B8] Colwell RK (2003) Biota 2: The Biodiversity Database Manager, + CD-ROM. Sinauer Associates, Sunderland, Massachusetts.

[B9] Flint OS Jr. (1998) Studies of Neotropical caddisflies, LIII: a taxonomic revision of the subgenus *Curgia* of the genus *Chimarra* (Trichoptera: Philopotamidae). Smithsonian Contributions to Zoology 594: 1-131. doi: 10.5479/si.00810282.594

[B10] Flint OS Jr. Sykora JL (2004) Caddisflies of Hispaniola, with special reference to the Dominican Republic (Insecta: Trichoptera). Annals of Carnegie Museum 73: 1-60.

[B11] Lago PK Harris SC (1987) The *Chimarra* (Trichoptera: Philopotamidae) of Eastern North America with descriptions of three new species. Journal of the New York Entomological Society 95: 226-251.

[B12] Santos APM Nessimian JL (2009) New species and records of *Chimarra* Stephens (Trichoptera, Philopotamidae) from Central Amazonia, Brazil. Revista Brasileira de Entomologia 53: 23-25. doi: 10.1590/S0085-56262009000100006

